# The Fascinating Flexibility and Coordination Modes of a Pentamethylene Connected Macrocyclic CNC Pincer Ligand

**DOI:** 10.3390/molecules26061669

**Published:** 2021-03-17

**Authors:** Ronja Jordan, Doris Kunz

**Affiliations:** Institut für Anorganische Chemie, Eberhard Karls Universität Tübingen, D-72076 Tübingen, Germany; Ronja.Jordan@uni-tuebingen.de

**Keywords:** pincer ligand, macrocycle, N-heterocyclic carbene, lithium, potassium, ruthenium, intramolecular C-H activation, dehydrogenation

## Abstract

The coordination chemistry of an electron-rich macrocyclic CNC pincer-ligand consisting of two pentamethylene tethered N-heterocyclic carbene moieties on a carbazole backbone (bimca^C5^) is investigated by mainly NMR spectroscopy and X-ray crystal structure analysis. A bridging coordination mode is found for the lithium complex. With the larger and softer potassium ion, the ligand adopts a facial coordination mode and a polymeric structure by intermolecular potassium nitrogen interactions. The facial coordination is also confirmed at a Cp*Ru fragment, while C-H activation under dehydrogenation at the alkyl chain is observed upon reaction with [Ru(PPh_3_)_3_Cl_2_]. In contrast, Pd(OAc)_2_ reacts under C-H activation at the central carbon atom of the pentamethylene tether to an alkyl-pincer macrocycle.

## 1. Introduction

Pincer-ligands, whose wingtips are tethered with simple or unfunctionalized hydrocarbon chains, are still very rare and an exception among phosphine pincer complexes [1,2]. For geometric reasons, bis(N-heterocyclic carbene) pincer ligands are more suitable for tethering. Notably, Chaplin and coworkers showed very interesting examples of macrocyclic CNC ligands that contain alkyl tethers between 8 and 16 C-atoms [3,4,5]. Their transition metal complexes showed particular properties depending on the ring size [6,7,8], but no reactivity at the tether itself.

Tethered CNC pincer complexes with smaller ring sizes are scarce [9], although potential ligand precursors are well known [10,11,12,13,14]. The interaction with the metal center should become stronger and influence its reactivity by steric restrictions.

When we reported on the improved synthesis of the so-called bimca ligand [15] (1,8-bis(imidazolin-2-ylidene)-3,8-di-*tert*-butylcarbazolide), a monoanionic CNC ligand, we also showed that a still relatively concentrated reaction mixture of 1,8-bis(imidazol-1-yl)-3,8-di-*tert*-butylcarbazol and 1,5-dibromopentane leads selectively to the bisimidazolium macrocycle **1a** (Hbimca^C5^)·2HBr [13]. However, until now, the coordination chemistry of this proligand remained unexplored. In the following, we will show that the tether leads not only to still unprecedented binding modes of the bimca ligand with alkali metals and to a suitable ligand for the facial coordination mode of the all sp^2^-hybridized framework, but that the proximity of the pentamethylene chain can also induce intramolecular C-H activation resulting in either dehydrogenation to an olefinic or formation of a carbanionic donor site.

## 2. Results and Discussion

### 2.1. Alkali Metal bimca^C5^ Complexes

The first step of the synthesis of metal complexes is the generation of the monoanionic carbene ligand bimca^C5^ with 3 equiv. of a strong base, preferentially an alkali metal base. As the N-H moiety is more acidic than the two imidazolium moieties, the independent generation of only the carbene moieties is not possible.

**Scheme 1:**
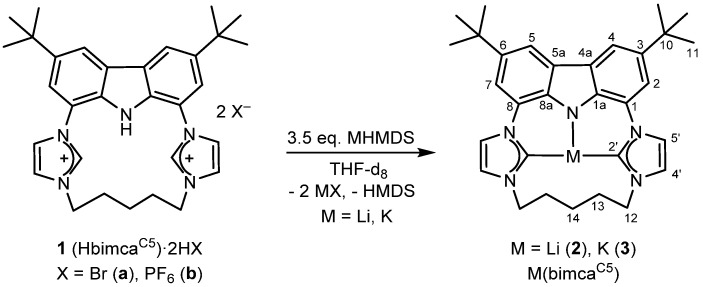
Three-fold deprotonation of the pentamethylene-tethered imidazolium salts **1** (Hbimca^C5^·2HX) leads to the formation of the alkali metal bimca^C5^ complexes **2** (Li) and **3** (K).

After combining the proligand **1b** with 3.5 equiv. of Li(N(SiMe_3_)_2_) in tetrahydrofuran, the successful formation of the desired lithium-pincer complex **2** (Scheme 1) can be optically recognized by the yellow color of the solution that shows a blue fluorescence at *λ* = 460 nm under UV light (330 nm), as has been observed qualitatively for the [Li(bimca^Me^)] complex before [15]. In the ^1^H NMR spectrum (THF-d_8_), the absence of the N-H and imidazolium C-H signals indicates full deprotonation, and thus, generation of the carbene moieties. In the ^13^C NMR spectrum, the carbene signal of the free NHC moiety would be expected at around 215 ppm. In our case, the signal at 204.5 ppm is characteristic for a coordination of the carbene moiety to lithium ions as it has been reported for other Li(bimca) complexes [13]. However, typically, the carbene signals of lithium complexes are shifted by about 20 ppm to higher field compared to the respective free carbenes. The carbene signal shows no direct ^13^C-^7^Li coupling, and in the ^7^Li NMR spectrum, only one broad signal at 0.31 ppm is observed at room temperature, which indicates a fast exchange of the lithium ions between the complex and solvated Li^+^ in solution on the NMR time-scale. We had to cool the sample down to −110 °C to determine the coupling constant ^1^*J*_CLi_ of about 24 Hz from the satellites of the Li signal at 2.06 ppm, which is separated at this temperature from the [Li(THF)_n_]^+^ signal at 0.65 ppm. At room temperature, the ^1^H NMR spectrum shows 3 signals in a 2:2:1 ratio for the methylene protons, which indicates fast conformational interconversion of the axial and equatorial ring protons. At −90 °C, two separate signals for the axial and equatorial protons of the N-CH_2_ moiety can be observed.

**Figure 1:**
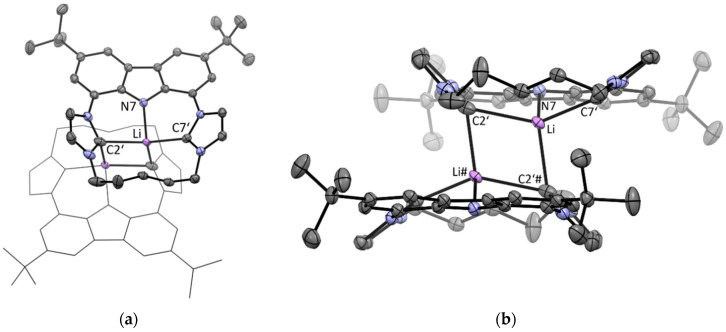
ORTEP-style plot of the molecular structure of [Li(bimca^C5^)] (**2**) in the solid state (anisotropic atomic displacement parameters at the 50% probability level). Hydrogen atoms are omitted for clarity. Multiple disordered solvent was removed applying the SQUEEZE routine. (**a**) Top view; the wireframe style of the symmetry equivalent lower part is used for clarity; (**b**) side view.

Single crystals suitable for X-ray structure analysis were obtained as a side product from the reaction of **2** with RuCl_2_ (vide infra). The molecular structure reveals a dimeric arrangement in the solid state in which the ligands are binding in a *κ*C,N,*κ*^2^C’ fashion, so that one Li-pincer complex forms a dimeric structure with a second one by a mutual bridging coordination of one carbene moiety each. Thus, a diamond-shape core with an inversion center results (Figure 1), in which the lithium carbon bond lengths measure Li-C2′ = 2.380(2) Å and Li-C2′# = 2.257(4) Å. The other coordinating bonds are pronouncedly shorter (Li-C7′ = 2.141(8) Å, Li-N = 1.965(5) Å). The formation of bridging Li(NHC) complexes with constrained geometry Cp-ligands has been recognized before [16].

It can be excluded that this dimeric structure is the prevailing structure in a solution in tetrahydrofuran, as it has a lower symmetry and would give rise to eight signals in the aromatic region of the ^1^H NMR spectrum, which is not observed.

**Figure 2:**
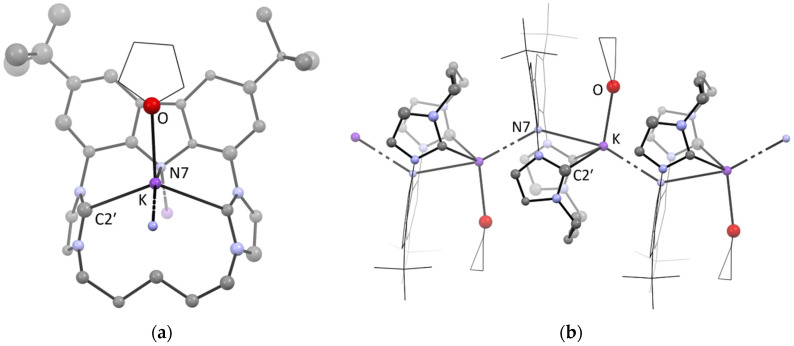
Two views of the isotropically refined structure of complex [K(bimca^C5^)] (**3**). For clarity, the wireframe style is used for the coordinated THF and the bimca framework where suitable. Co-crystallized solvent and hydrogen atoms have been omitted for clarity: (**a**) view along the two-fold axis polymer chain with one monomer depicted; (**b**) side view of the coordination polymer. The K(bimca^C5^)(THF) moieties are additionally coordinated by the carbazole nitrogen atom of the next complex that is rotated by 180° in a zig-zag fashion, thus forming a coordination polymer along a two-fold axis.

Crystallographically characterized lithium complexes with NHC pincer or tripodal NHC complexes are very rare [17]. Due to the preferred tetrahedral coordination sphere as well as the small ion radius of the lithium ion, a symmetric T-shape pincer coordination seems not favorable. To probe whether larger alkali metal ions lead to the typical meridional coordination of the bimca ligand, we deprotonated the proligand **1** with 3 equiv. of KHMDS (potassium hexamethyldisilylamide) in THF-*d*_8_. The blue fluorescence indicates full deprotonation, but vanishes upon darkening of the solution and precipitation of the complex **3**. Nevertheless, NMR spectra could be obtained from the diluted solution. The ^1^H NMR spectrum shows the signal pattern of a symmetric complex and fast conformational changes in the alkyl chain. The four aromatic signals are upfield-shifted in comparison to lithium complex **2**, especially the signals of the imidazole backbone. This can be explained with the reduced Lewis-acidity of the potassium ion [18,19,20,21]. The reduced influence of the Lewis acid is also reflected in the upfield shift of the methylene signals H-12 and H-13 of the dangling alkyl tether, while the signal of H-14 is not affected. In the ^13^C NMR spectrum, the carbene signal at 213.1 ppm lies very close to that of a comparable free carbene (~215 ppm) [19], which confirms the very weak binding of the potassium ion. From the filtrate we obtained small crystals that were subjected to X-ray structure analysis. The limited quality of the data due to multiple twinning and inclusion of powder in the crystal allows no discussion of bond lengths and angles, but the results confirm unambiguously the coordination mode of the ligand (Figure 2). The potassium is coordinated by five donor atoms. The bimca^C5^ ligand coordinates in a facial manner instead of the typical meridional pincer fashion. The nitrogen donor is not coordinating within the carbazole plane, but the potassium is oriented almost perpendicular to the plane. The carbazole nitrogen coordinates to the potassium center of the next complex monomer, which is oriented by a rotation of 180°, thus forming a polymeric structure along this two-fold axis with almost equidistant K-N bonds (3.0 Å) in a zig-zag fashion. The fifth coordination site is occupied by tetrahydrofuran.

### 2.2. Ruthenium(II) bimca^C5^ Complexes

#### 2.2.1. Facial Coordination of the bimca^C5^ Ligand

The facial coordination mode is untypical for fully sp^2^ hybridized pincer ligands. An anionic CNP pincer ligand was reported to form a dimeric facially coordinated potassium complex in the solid state [22]. A dimeric K[Pd(bimca^Me^)] complex shows additional facial coordination to the potassium counterion [23].

**Scheme 2:**
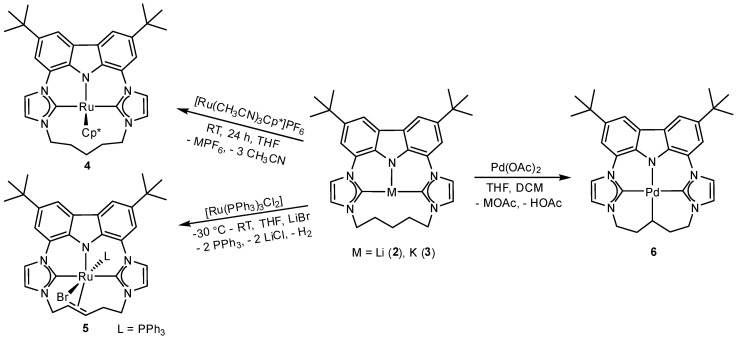
Formation of Ru(II) and Pd(II) complexes with different coordination modes of the bimca^C5^ ligand from the in situ-generated alkali metal complexes **2** (Li) and **3** (K).

We had shown earlier that the *N*-homoallyl substituted bimca ligand bimca^Homo^ is able to coordinate in a facial mode when the in situ-generated [Li(bimca^Homo^)] is reacted with [Ru (NCCH_3_)_3_Cp*]PF_6_ [24]. The macrocyclic ligand bimca^C5^ should be even more suitable for this purpose due to the C5-tether. Therefore, we reacted [Li(bimca^C5^)] (**2**) with the ruthenium precursor and obtained the desired product complex **4** in 84% yield as a red solid (Scheme 2).

**Figure 3:**
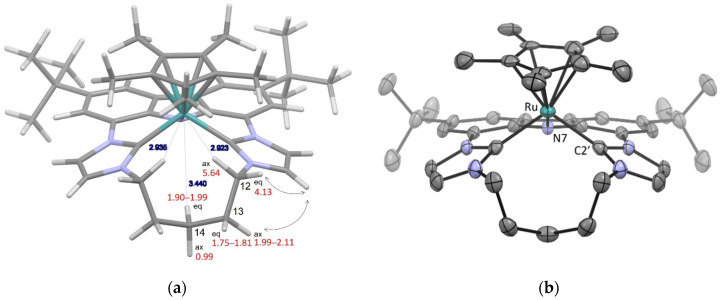
Molecular structure of complex **4**. (**a**) DFT-optimized structure showing the chemical shifts of the methylene signals (red) and important NOE cross peaks (black arrow) as well as the distance between the metal and close hydrogen atoms (blue); (**b**) ORTEP-style plot of the molecular structure of complex **4** (anisotropic atomic displacement parameters at the 50% probability level). Hydrogen atoms are omitted for clarity. The Cp* ligand is highly disordered.

In the ^1^H NMR spectrum, the four signals of the aromatic bimca protons indicate a *C*_s_ symmetry. With the help of 2D NMR spectra, including an NOE experiment as well as comparing the coupling constants with a DFT-optimized structure (which is almost identical to the molecular structure obtained by X-ray structure analysis (vide infra)), the six signals of the diastereotopic methylene protons can be assigned. The chemical shift difference between the signals of those protons pointing towards the metal center vs. those pointing away amounts to Δδ = 1.5 ppm in case of the NCH_2_ signals (H-12) and still Δδ = 1.0 ppm for those of H-14 (Figure 3a). As anagostic interactions can be excluded due to the long H⋯Ru distances (>2.9 Å) [25], this can be explained with the magnetic effect of the metal.

From a concentrated solution of **4** in THF/diethylether at room temperature, red plates were obtained that were suitable for X-ray single crystal structure analysis. The facial binding mode of the bimca^C5^ ligand is confirmed (Figure 3b) and the overall geometry is similar to that reported for the bimca^Homo^ analogue [24]. The Ru atom is located 1.135 Å over the plane spanned by the donating carbene and carbazole nitrogen atoms.

The macrocyclic ligand should be ideal to avoid the formation of octahedral complexes with two meridionally coordinating pincer ligands, which is typically observed when reacting monoanionic pincer ligands with RuCl_2_ or with FeCl_2_ or IrCl(PPh_3_)_3_ under oxidation [26]. We also tried to obtain the ruthenocene analogue Ru(bimca^C5^)_2_ with two facially coordinating bimca^C5^ ligands instead of one and one Cp* ligand, but reacting [Li(bimca^C5^)] with RuCl_2_ did not result in a defined compound.

#### 2.2.2. Complex Formation under Dehydrogenation of bimca^C5^

When we reacted [Ru(PPh_3_)_3_Cl_2_] with a freshly prepared solution of the potassium complex **3** in THF at −30 °C, red crystals formed after 24 h (Scheme 2). The product is only poorly soluble in THF, slightly soluble in dichloromethane, soluble in methanol and decomposes in DMSO. In the ^1^H NMR spectrum the signal pattern indicates a mixture of at least two asymmetric complexes in a 1:0.4 ratio. The two multiplets at 5.20–5.27 (H-14) and 5.07–5.10 ppm (H-13) indicate the formation of a double bond, which is corroborated by the respective ^13^C NMR signals at 81.6 (C13) and 88.4 (C14). All remaining signals in the alkyl region can be assigned to the six non-equivalent hydrogen atoms as well as the carbon signals by means of ^1^H,^13^C correlation spectroscopy and NOE experiments. Therefore, a dehydrogenation under C-H activation must have occurred so that complex **5** is the final reaction product. The formation of H_2_ is not observed in the ^1^H NMR spectrum; however, this might be the case due to its volatility. In methanol, a qualitative similar ^1^H NMR spectrum is obtained. The peaks of the second isomer are partly covered by those of the main isomer. In the ^31^P NMR spectrum two very close peaks at 64.3 and 67.8 ppm indicate the presence of two isomers of complex **5** with a similar constitution. The formation of a carbene complex by double C-H activation at the central carbon atom C14, as was observed with a pentenyldiphosphine ruthenium complex by Gusev in low amounts [27], was not observed in our case.

**Figure 4:**
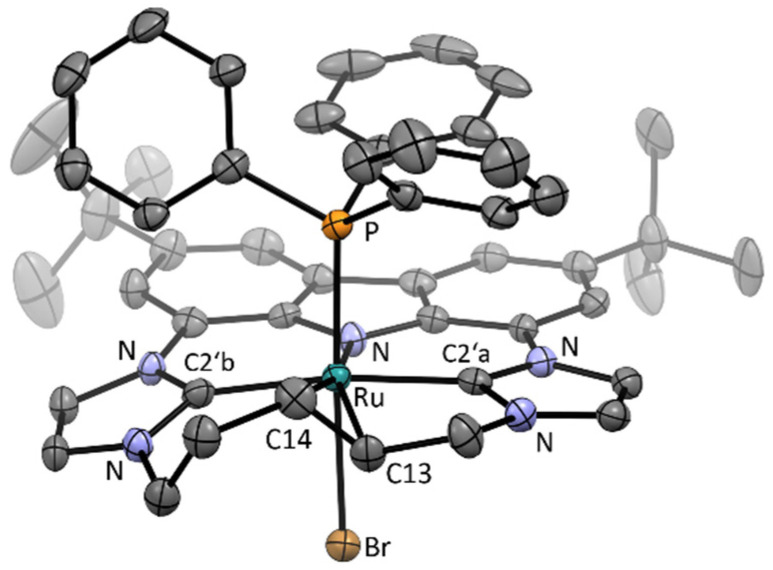
ORTEP-style plot of the molecular structure of complex [Ru(bimca^C5H8^)Br(PPh_3_)] (**5**) (anisotropic atomic displacement parameters at the 50% probability level). Hydrogen atoms and two molecules of dichloromethane are omitted for clarity.

The red crystals were suitable for X-ray structure analysis. The molecular structure is depicted in Figure 4 and confirms the formation of a double bond that is coordinated to the Ru center at a distance of 2.208 (Ru-C13) and 2.284 Å (Ru-C14). A disorder in the alkyl chain prevents the discussion of C-C bond lengths. The Ru-Carbene bonds and the Ru-N bond are in a typical range. Due to the octahedral coordination mode and the non-symmetric dehydrogenated C5 chain, it becomes apparent that the other isomer could be the diastereomer obtained by inversion of the phosphine and bromido ligand.

While C-H activation of arenes by Ru complexes is well established since its first mentioning by Chatt in 1962 [28,29] and the successive developments based on the Murai reaction [30,31,32], the inter- and intramolecular C-H activation of alkanes by Ru complexes is still rare [26,27]. The first observed cyclometallation was reported by Chatt from a Ru(0) intermediate in 1965 [29,33]. In the past few years, the dehydrogenation of alkanes by a Ru(0) catalyst was reported by Goldman [34] as well as its acceptorless variant with Ru(II) pincer catalysts by Roddick and Huang [35,36].

### 2.3. A macrocyclic Palladium(II) bimca^C5^ Complex by C-H Activation

To probe on a small scale whether Pd(II), which usually forms square planar complexes, is also suitable for an intramolecular C-H activation at room temperature, we reacted the freshly generated [Li(bimca^C5^)] in tetrahydrofuran with Pd(II) acetate (Scheme 2). The reaction mixture turned brownish and was left overnight at −30 °C. The solvent was removed in vacuo and the residue washed with pentane to obtain a light brown residue after drying.

**Figure 5:**
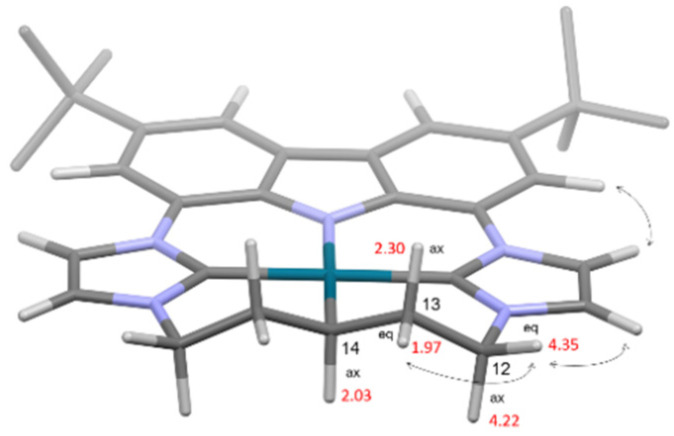
DFT (BP86/def2-TZVP)-optimized structure of [Pd(bimca^C5H9^)] (**6**), showing the chemical shifts of methylene and methine signals (red) and important NOE cross peaks (black arrow).

The ^1^H-NMR spectrum in dichloromethane-d_2_ shows the symmetry reduced signal set with four aromatic signals and no typical signals of an olefin. The pentamethylene chain leads to five signals at 4.35 (12-H_eq_), 4.22 ppm (12-H_ax_), 2.30 (13-H_ax_), 2.03 (14-H_ax_) and 1.97 (13-H_eq_) in a 2:2:2:1:2 ratio, which can be assigned based on the characteristic coupling pattern to the equatorial and axial protons (Figure 5). The diastereotopic signals also confirm the conformational stability of the pentamethylene chain that can be explained by a C-H activation at the C14 position under formation of a tetradentate macrocyclic ligand. In the ^13^C NMR spectrum, the carbene signal is detected at 171.0 ppm and the Pd-C14 signal at 23.8 ppm. The latter is slightly shifted upfield compared to the signal of an NCN pincer ligand based on 1,5-bis(pyrazol-1-yl)pentane (δ = 2.54 (^1^H)/28.4 (^13^C)) [37]. In the HR-ESI mass spectrum, the molecule peak with its characteristic Pd isotope pattern is superimposed by the [M+H]^+^ peak.

The C-H activation of an alkyl chain which is close to a Pd(II) center is well known from the formation of PCP or NCN pincer complexes from the reaction of Pd(II) acetate with 1,5-bis(phosphino)pentane in refluxing ethanol [38] or with 1,5-bis(N-heterocycle)pentane in refluxing acetic acid [37]. The ease of the C-H activation in our case can be rationalized with the proximity of the pentamethylene chain at the already-formed CNC pincer complex with an intramolecular deprotonation by the acetate ligand (concerted metalation deprotonation (CMD) mechanism) [39].

## 3. Materials and Methods

All reactions were carried out under argon atmosphere in dried and degassed solvents with Schlenk technique or in a glovebox (MBraun-Labmaster). Chemicals for synthesis were commercially available. Solvents were purchased from Sigma Aldrich and dried with an MBraun SPS-800 solvent purification system and degassed. Young NMR tubes from Deutero were used for measuring air- and water-sensitive products. NMR spectra were recorded using a Bruker AVANCE II+ 400 spectrometer or a Bruker AVANCE AVII+ 500. The chemical shifts (*δ*) are reported in [ppm] and the ^1^H NMR spectra are referenced to the residual protonated solvent peak: *δ*_H_ (THF-*d*_7_) = 1.72 and 3.57 ppm, *δ*_H_ (DMSO-*d*_5_) = 2.50 ppm, *δ*_H_ (CDHCl_2_) = 5.32 ppm and *δ*_H_ (CD_2_HCN) = 1.94 ppm. ^13^C{^1^H} NMR spectra are referenced to the signal of the deuterated solvent: *δ*_C_ (THF-*d*_8_) = 25.2 und 67.4 ppm, *δ*_C_ (CD_2_Cl_2_) = 53.8 ppm [40,41]. The following abbreviations were used: s = singlet, d = doublet, *t* = triplet, m = multiplet, *p* = pseudo and variations of them. Coupling constants (*J*) are expressed in [Hz]. 2D NMR correlation spectra were used for peak assignment. Mass spectra were measured on an APEX FT-ICR of Bruker und Daltonik Maxis 4G. UV/VIS spectra were collected using a Jasco V-770 UV-Visible/NIR spectrophotometer. Fluorescence spectra were recorded with a PTI Quantamaster QM4 spectrofluorometer equipped with a 75 W continuous xenon short arc lamp as an excitation source. The emission was monitored using a monochromator at 1200 grooves/mm and detected with a PTI P1.7R detector module (Hamamatsu PMT R5509-72 with a Hamamatsu C9525 power supply operated at 1500 V). Elemental analysis was carried out on a varioMICRO V1.9.2 of Elementar Analysensysteme GmbH. The measurements were recorded in the CHNS modus.

X-ray diffraction data were collected on a Bruker APEX Duo CCD with an Incoatec I*µ*S microfocus sealed tube and QUAZAR optics for Mo*K*_α_ radiation (*λ* = 0.71073 Å). Corrections for absorption effects were applied using SADABS or TWINABS. All structures were solved by direct methods using the ShelXle [42,43,44] software package for structure solution and refinement. In the case of structures **2** and **4** the SQEEZE routine was applied [45]. CCDC 2,064,676 (**3**), 2,064,677 (**4**), 2,064,678 (**5**) and 2,064,679 (**2**) contain the supplementary crystallographic data of this paper (Supplementary Materials). These data are provided free of charge by the joint Cambridge Crystallographic Data Centre and Fachinformationszentrum Karlsruhe Access Structures service, www.ccdc.cam.ac.uk/structures (accessed on 21 January 2021).

Calculations were performed based on density functional theory at the BP86/def2-TZVP [46,47,48,49,50,51] level implemented in Turbomole [52,53,54,55,56,57,58,59,60,61]. The RI-approximation [62,63,64,65,66,67] was used all over as well as the Grimme dispersion correction [68,69]. The cartesian coordinates of the geometry optimized structures are available as xyz-file in the Supporting Information.

### 3.1. Preparation of (Hbimca^C5^)·2HPF_6_ (***1b***)

1 eq (Hbimca^C5^)·2HBr (**1a**) [13] was stirred in water for 20 min at 60 °C. Afterward, the suspension was filtered and 1 eq KPF_6_ was added. Upon stirring for 2 h, the colorless product (Hbimca^C5^)·2HPF_6_ (**1b**) precipitated and was filtered off, washed with water and *n*-hexane, and dried in vacuo. The yields were usually about 30%. The ^1^H NMR-spectroscopic data are comparable to those of compound **1a** [13]. Deviations in the chemical shifts of the signals of the acidic protons can be due to changes in hydrogen bonding of the different counterion as well as to variable concentrations of residual water from the deuterated solvent, which is a known phenomenon [13].

^1^H NMR (400.11 MHz, DMSO-d_6_): *δ* = 1.46 (s, 18H, H-11), 1.66–1.71 (m, 2H, H-14), 1.77–1.83 (m, 4H, H-13), 4.33–4.36 (m, 4H, H-12), 7.79 (d, ^4^*J*_HH_ = 1.1 Hz, 2H, H-2/7), 8.05 (br s, 2H, H-4‘), 8.27 (br s, 2H, H-5‘), 8.60 (d, ^4^*J*_HH_ = 1.1 Hz, 2H, H-4/5), 9.65 (s, 2H, H-2‘), 11.18 (s, 1H, NH). ^19^F NMR (376.48 MHz, DMSO-d_6_): 70.2 (d, ^1^*J*_PF_ = 711.4 Hz, PF_6_).

### 3.2. General Procedure for the Generation of Alkali Metal bimca^C5^ Complexes

A suspension of proligand **1** (Hbimca^C5^)·2HX (X = Br (**a**), PF_6_ (**b**)) and 3.5 equiv. of MHMDS (M = Li, K) were stirred in 0.5 mL THF-d_8_ for 5 min.

Complex **2** (Li(bimca^C5^)): A yellow solution with a blue fluorescence under UV-light was obtained. ^1^H NMR (400.11 MHz, THF-d_8_): *δ* 1.50 (s, 18H, H-11), 1.85–1.90 (m, 2H, H-14), 2.07–2.13 (m, 4H, H-13), 4.11–4.13 (m, 4H, H-12), 7.05 (d, ^3^*J*_HH_ = 1.7 Hz, 2H, H-4‘), 7.35 (d, ^4^*J*_HH_ = 1.7 Hz, 2H, H-2/7), 7.52 (d, ^3^*J*_HH_ = 1.7 Hz, 2H, H-5′), 7.99 (d, ^4^*J*_HH_ = 1.7 Hz, 2H, H-4/5). ^13^C{^1^H} NMR (100.61 MHz, THF-d_8_): *δ* 23.9 (C14), 30.4 (C13), 33.0 [69] (C11), 35.3 (C10), 50.3 (C12), 111.4 (C2/7), 114.7 (C4/5), 119.9 (C4′ or C5‘), 120.2 (C4′ or C5‘), 127.7 (C4a/5a), 129.0 (C1/8), 135.5 (C3/6), 144.9 (C1a/8a), 204.4 (C2′). ^7^Li NMR (194.37 MHz, THF-d_8_): 2.06 (Li(bimca^C5^)), 0.65 (LiHMDS/LiX). UV/VIS (THF): *λ*_1_ = 246 nm (ε = 8·10^3^ L·mol^−1^·cm^−1^), *λ*_2_ = 300 nm (ε = 5·10^3^ L·mol^−1^·cm^−1^), *λ*_3_ = 347 (ε = 2·10^3^ L·mol^−1^·cm^−1^). Fluorescence (THF): *λ* = 460 nm (ex: 330 nm).

Complex **3** (K(bimca^C5^)): ^1^H NMR (400.11 MHz, THF-d_8_): *δ* 1.45 (s, 18H, H-11), 1.62–1.69 (m, 4H, H-13), 1.85–1.96 (m, 2H, H-14), 4.13 (pst, ^3^*J*_HH_ = 4.7 Hz, 4H, H-12), 6.83 (s, br, 2H, H-4′), 6.96 (s, br, 2H, H-5′), 7.16 and 7.99 (each d, each ^4^*J*_HH_ = 1.8 Hz, each 2H, H-4/5, H-2/7). ^13^C{^1^H} NMR (100.61 MHz): *δ* 32.9 (C11‘), 33.5 (C13), 35.0 (C10), 52.3 (C12), 114.7 (C2/7 or C4/5), 115.0 (C2/7 or C4/5), 117.7 (C4‘), 122.7 (C5′), 127.7 (C1/8), 130.7 (C4a/5a), 133.9 (C3/6), 148.1 (C1a/8a), 212.5 (C2‘). The peak of C14 was not detected.

### 3.3. Synthesis of the Ru(II) Sandwich Complex ***4*** (Ru(bimca^C5^)Cp*)

A total of 40 mg (51.8 µmol, 1 eq.) of the proligand **1b** (X = PF_6_) and 33.1 mg (165 µmol, 3.2 equiv.) KHMDS were mixed in 1 mL tetrahydrofuran. After five minutes, the reaction was completed and the mixture cooled to −30 °C. The ruthenium precursor (24.0 mg, 51.6 µmol, 1 equiv.) [Ru(CH_3_CN)_3_Cp*]PF_6_ was added to the solution under further cooling and stirring. Upon dissolving of the precursor, the solution was warmed to room temperature. After stirring for 24 h, the solvent was removed in vacuo and the remaining solid extracted with *n*-pentane. The extract was dried again in vacuo to obtain the product as a red solid in 28 mg (84%) yield. ^1^H NMR (400.11 MHz, THF-d_8_): *δ* 0.99 (ps dd, ^2^*J*_HH_ = 14.3 Hz, ^3^*J*_HH_ = 6.6 Hz, 1H, H-14_ax_), 1.05 (s, 15H, Cp*), 1.47 (s, 18H, H-11), 1.75–1.81 (m, 2H, H-13_eq_), 1.90–1.99 (m, 1H, H-14_eq_), 1.99–2.11 (ps dt, 2H, ^2,3^*J*_HH_ = 13.8 Hz, ^3^*J*_HH_ = 6.6 Hz, H-13_ax_), 4.13 (ps d, ^2^*J*_HH_ = 13.3 Hz, 2H, H-12_eq_), 5.64 (ps t, ^2,3^J_HH_ = 13.2 Hz, 2H, H 12_ax_), 7.24 (d, ^4^*J*_HH_ = 0.8 Hz, 2H, H-2/7), 7.25 (d, ^3^*J*_HH_ = 2.1 Hz, 2H, H-4′), 7.71 (d, ^4^*J*_HH_ = 0.8 Hz, 2H, H-4/5), 7.91 (d, ^3^*J*_HH_ = 2.2 Hz, 2H, H-5′). ^13^C{^1^H} NMR (100.61 MHz, THF-d_8_): *δ* = 10.2 (Cp*-*C*H_3_), 16.8 (C14) 33.0 (C11), 35.4 (C13), 35.6 (C10), 51.0 (C12), 83.4 (Cp*), 106.6 (C2/7), 114.2 (C4/5), 119.1 (C5′), 120.6 (C4‘), 126.9 (C4a/5a), 129.2 (C1/8), 137.7 (C3/6), 143.4 (C1a/8a), 196.2 (C2′). MS (ESI^+^, THF/CH_3_CN, *m/z*) = 715.1 [C_41_H_51_N_5_Ru+H]^+^, 952.1 [C_41_H_51_N_5_Ru+Ru+Cp*]^+^. MS (HR-ESI^+^, CH_3_CN) calcd for [C_41_H_51_N_5_Ru]^+^ 715.31825; found 715.31911, relative mass deviation = 0.34 ppm.

### 3.4. Synthesis of the Macrocyclic Ru(II)(bimca^C5^) Complex ***5***

A total of 20.3 mg (31.6 µmol, 1 eq.) (Hbimca^C5^)·2HBr (**1a**) and 20.4 mg (94.8 µmol, 3.3 eq) KHMDS were stirred in 2 mL tetrahydrofuran. The reaction was completed after five minutes and 30.0 mg (31.6 µmol, 1 eq.) [Ru(PPh_3_)_3_Cl_2_] was added to the reaction mixture. After stirring for an additional five minutes, the mixture was stored for 24 h at −30 °C, whereupon the product precipitated as red crystals. They were filtered off and washed with *n*-pentane and diethyl ether 1 mL each. The NMR spectra show the formation of a mixture of two isomers in a 1:0.4 ratio.

Major isomer: ^1^H NMR (400.11 MHz, dichloromethane-d_2_): *δ* 1.45 (s, 18H, H-11), 1.93–2.03 (m, 1H, H-15), 3.29–3.34 (m, 1H, H-15), 4.09–4.13 (m, 1H, H-16), 4.43–4.55 (m, 2H, H-16, H-12), 4.75–4.81 (m, 1H, H-12), 5.07–5.10 (m, 1H, H-14), 5.20–5.27 (m, 1H, H-13), 7.29–7.35 (m, PPh_3_), 7.38 (s, br, 1H, H-4‘), 7.46–7.50 (m, PPh_3_), 7.55–7.59 (m, PPh_3_), 7.65 (s, br, 1H, H-2/7 or H-4/5), 7.63–7.70 (m, PPh_3_), 7.76 (s, br, 1H, H-2/7 or H-4/5), 7.73 (s, br, 1H, H-4‘), 7.98 (s, br, 1H, H-5‘), 8.16 (s, 1H, H-2/7 or H-4/5), 8.17 (s, 1H, H-2/7 or H-4/5), 8.29 (s br, 1H, H-5‘). Signal H-4′ is superimposed by the PPh_3_ signal at 7.29–7.35 ppm. ^13^C{^1^H} NMR (100.61 MHz, dichloromethane-d_2_): *δ* 31.3 (C15), 32.5 (C11), 32.5 (C11), 35.2 (C10), 35.3 (C10), 51.5 (C12 or C16), 52.1 (C12 or C16), 81.6 (C13), 88.4 (C14), 109.8 (C2/7), 111.4 (C2/7), 115.0 (C4/5), 115.1 (C4/5), 117.0 (C5′), 118.3 (C5′), 119.6 (C4′), 123.9 (C4′), 127.5 (C4a/5a), 127.8 (C4a/5a), 129.0 (CPPh_3_), 129.1 (CPPh_3_), 132.4–132.5 (CPPh_3_), 134.1 (CPPh_3_), 134.3 (CPPh_3_), 135.8 (C1a/8a), 139.2 (C3/6), 139.5 (C-3/6). Signal C2′ was not detected and signal C1/8 is superimposed by the PPh_3_ signal at 129.0–192.1 ppm.

Minor isomer: 1.55 (s, 18H, t-Bu), 2.64–2.73 (m, 1H, C_5_H_8_), 3.09–3.11 (m, 2H, C_5_H_8_), 3.53–3.58 (m, 1H, C_5_H_8_), 4.43–4.55 (m, 2H, C_5_H_8_), 4.98–5.04 (m, 1H, C_5_H_8_), 5.75–5.28 (m, 1H, C_5_H_8_), 7.29–7.33 (PPh_3_), 7.40 (s br, 1H, ArH), 7.46–7.48 (PPh_3_), 7.56–7.59 (PPh_3_), 7.67–7.70 (m, PPh_3_), 7.73 (s br, 1H, ArH), 8.06 (s br, 1H, ArH), 8.16–8.18 (2H, ArH), 8.25 (s br,1H, ArH). Two aromatic signals were not detected.^31^P{^1^H} NMR (MeOD-d_4_, 161.97 MHz): δ = 67.8 (1P, PPh_3_), 64.3 (0.4P, PPh_3_, minor isomer). MS (ESI^+^, MeOH, *m/z*) = 840.2 (100) [C_49_H_49_BrN_5_PRu-Br]^+^, 480.3 (10) [ligand]^+^. CHN: (C_49_H_49_BrN_5_PRu): Calcd C 63.98, H 5.37, N 7.61 found C 63.74, H 5.63, N 7.33.

### 3.5. Preparation of the Macrocyclic Pd(II) Complex ***6***

A total of 30.0 mg (46.8 µmol, 1 eq) (Hbimca^C5^)·2HBr (**1**) and 27.4 mg (164.4 µmol, 3.5 eq) LiHMDS were mixed and dissolved in 2 mL of tetrahydrofuran. Moreover, 10.5 mg (46.8 µmol, 1 eq) [Pd(OAc)_2_] were added and the reaction mixture was kept at −30 °C for 24 h. After warming to room temperature, the solvent was removed in vacuo and the residue washed three times with 1 mL *n*-pentane. The product is a pale brown solid that is insoluble in tetrahydrofuran and soluble in dichloromethane.

^1^H NMR (400.11 MHz, dichloromethane-d_2_): *δ* 1.53 (s, 18H, H-11), 1.97 (d ps t, ^3^*J*_HH_ ~ 2.6 Hz, ^2/3^*J*_HH_~13.8 Hz, 2H, H-13_eq_), 2.03 (tt, ^3^*J*_HH_ = 2.6 Hz, ^3^*J*_HH_ = 12.0 Hz, 1H, H-14_ax_), 2.30 (ps qd, ^2/3^*J*_HH_ ~ 12.9 Hz, ^3^*J*_HH_ = 2.8 Hz, 2H, H-13_ax_), 4.22 (pst d, ^2/3^*J*_HH_ = 12.5 Hz, ^3^*J*_HH_ = 1.7 Hz, 2H, H-12_ax_), 4.35 (d ps t, ^2^*J*_HH_ = 12.6 Hz, ^3^*J*_HH_ = 3.3 Hz, 2H, H-12_eq_), 7.13 (d, ^3^*J*_HH_ = 1.9 Hz, 2H, H-4‘), 7.69 (d, ^4^*J*_HH_ = 1.4 Hz, 2H, H-2/7), 8.08 (d, ^3^*J*_HH_ = 1.9 Hz, 2H, H-5‘), 8.14 (d, ^4^*J*_HH_ = 1.4 Hz, 2H, H-4/5). ^13^C{^1^H} NMR (100.61 MHz, dichloromethane-d_2_): *δ* 23.8 (C14), 32.7 (C11), 35.4 (C10), 38.3 (C13), 53.4 (C12), 110.1 (C2/7), 114.8 (C4/5), 115.8 (C5‘), 122.3 (C4‘), 124.6 (C1/8), 126.7 (C4a/5a), 135.7 (C1a/8a), 138.6 (C3/C6), 171.0 (C2′). MS (HR-ESI^+^, CH_3_CN) calcd for [C_31_H_35_N_5_Pd]^+^ = 583.19272; found 583.19679, relative mass deviation = 6.98 ppm; calcd for [C_31_H_35_N_5_Pd+H]^+^ 584.20001, found 584.19985, relative mass deviation = 2.27 ppm.

## 4. Conclusions

We have shown that the introduction of a pentamethylene tether into a CNC pincer ligand leads to a rich coordination chemistry and an increase of possible coordination modes. Due to the proximity of the alkyl tether at the metal center, C-H activation reactions were observed, which—depending on the nature of the metal and additional ligands—can lead to a dehydrogenation (Ru) or a deprotonation (Pd). Thus, the bimca^C5^ ligand can not only serve as a monoanionic pincer ligand, but also as a monoanionic (bimca^C5H8^) as well as a dianionic (bimca^C5H9^) tetradentate macrocyclic ligand. In addition, the hindered rotation of the carbene moieties in the tethered pincer ligand enhances a facial coordination mode (Ru, K). From this point of view, the formation of a dimeric lithium complex by a chelating and bridging coordination mode is rather unexpected and likely due to the small size of the lithium ion. 

Especially the hapticity increase of the bimca^C5^ ligand leads to new options for the catalyst design as the ligand could play an active role in the activation of substrates or stabilize highly reactive intermediates. Studies on these aspects will be the topic of future research.

## Data Availability

The data is available in the Supplementary Material.

## Supplementary Materials

The following are available online: SI containing the spectra of all new compounds, crystallographic information and the xyz-file of the calculated structures.

## Sample Availability

Samples of the compounds might be available from the authors.
