# Supplementary material for: The Fascinating Flexibility and Coordination Modes of a Pentamethylene Connected Macrocyclic CNC Pincer Ligand
Source: Molecules. 2021 Mar 17;26(6):1669. doi: 10.3390/molecules26061669 (PMC8002578; doi:10.3390/molecules26061669)
Supplement: Supplementary file 1 [file molecules-26-01669-s001.zip › Si_Revised.pdf]

Supporting Information

The fascinating flexibility and coordination modes of a pentamethylene connected macrocyclic CNC pincer ligand

Ronja Jordan [1], and Doris Kunz [1,*]

[1] Eberhard Karls Universität Tübingen; Doris.Kunz@uni-tuebingen.de
* Correspondence: Doris.Kunz@uni-tuebingen.de; +49-7071-29-72063

Table of Contents

1 NMR-Spectroscopy 2

2 HR-MS Spectrum of (6) 37

3 Crystal structure analysis 38

4 UV-Vis and Fluorescence Spectroscopy 39

1 NMR-Spectroscopy

(Hbimca^{C5})·2HPF$_6$ (1b):

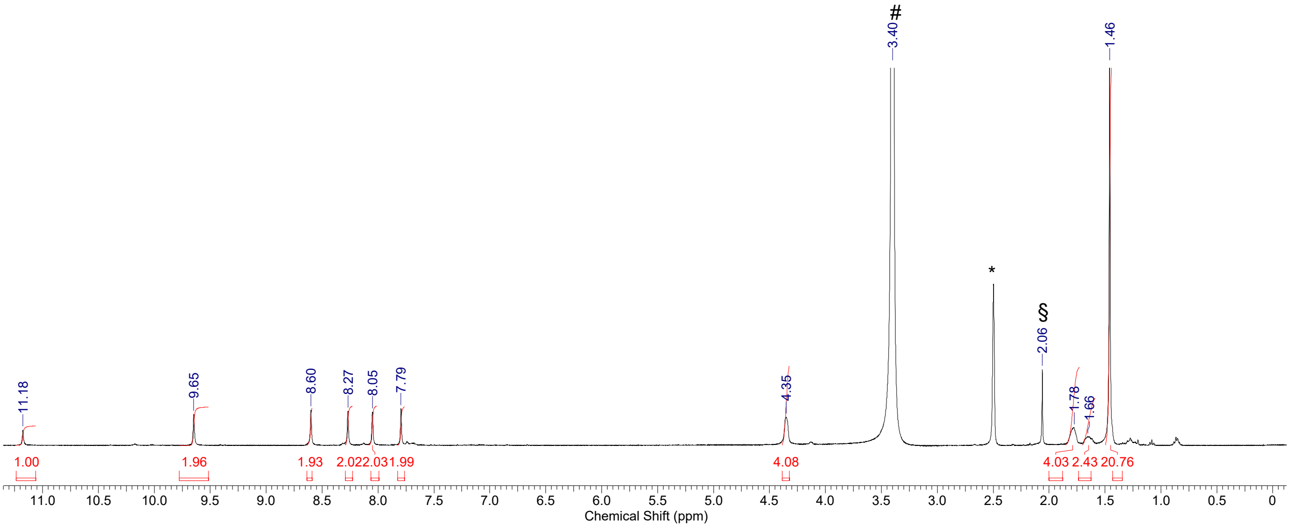

*Figure 1: ^{1}H NMR spectrum (400 MHz, DMSO-d$_6$ (*)) of (Hbimca^{C5})·2HPF$_6$ (**1b**) (MeCN (§), water (#)).*

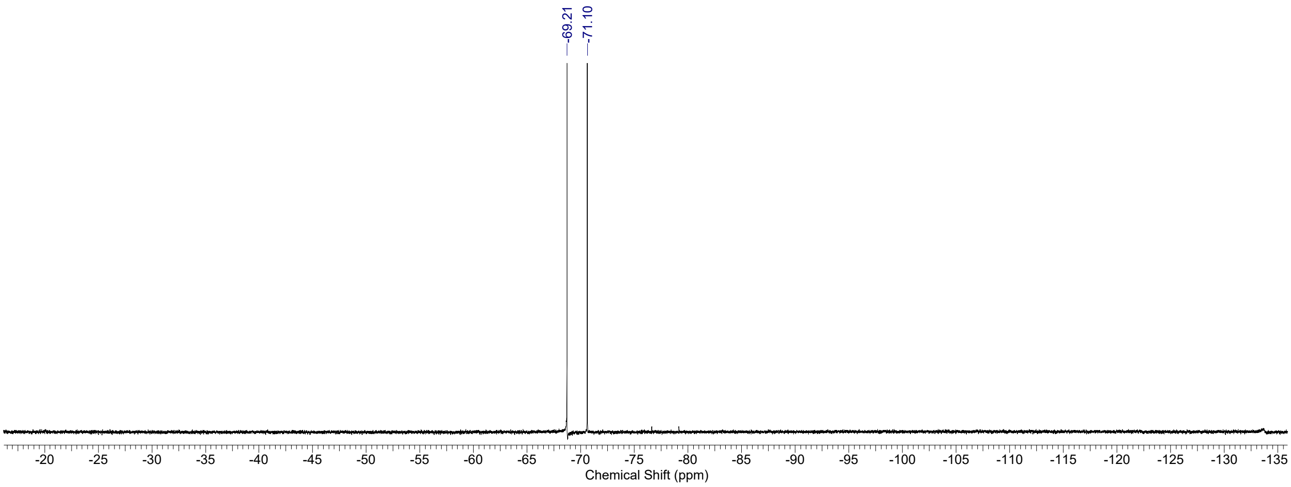

*Figure 2: ^{19}F NMR spectrum (376.48 MHz, DMSO-d$_6$ (*)) of (Hbimca^{C5})·2HPF$_6$ (**1b**).*

[Li(bimca^{C5})] (2):

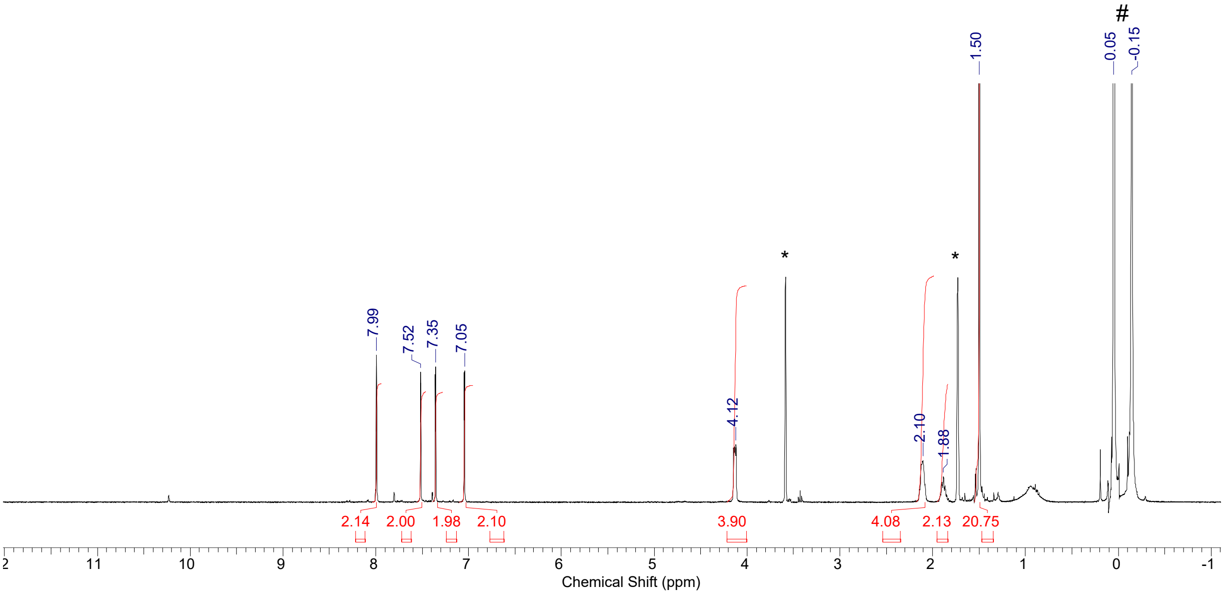

*Figure 3: ^{1}H NMR spectrum (400 MHz, THF-d$_8$ (*)) of [Li(bimca^{C5})] (**2**) generated with LiHMDS (#); HMDS (#).*

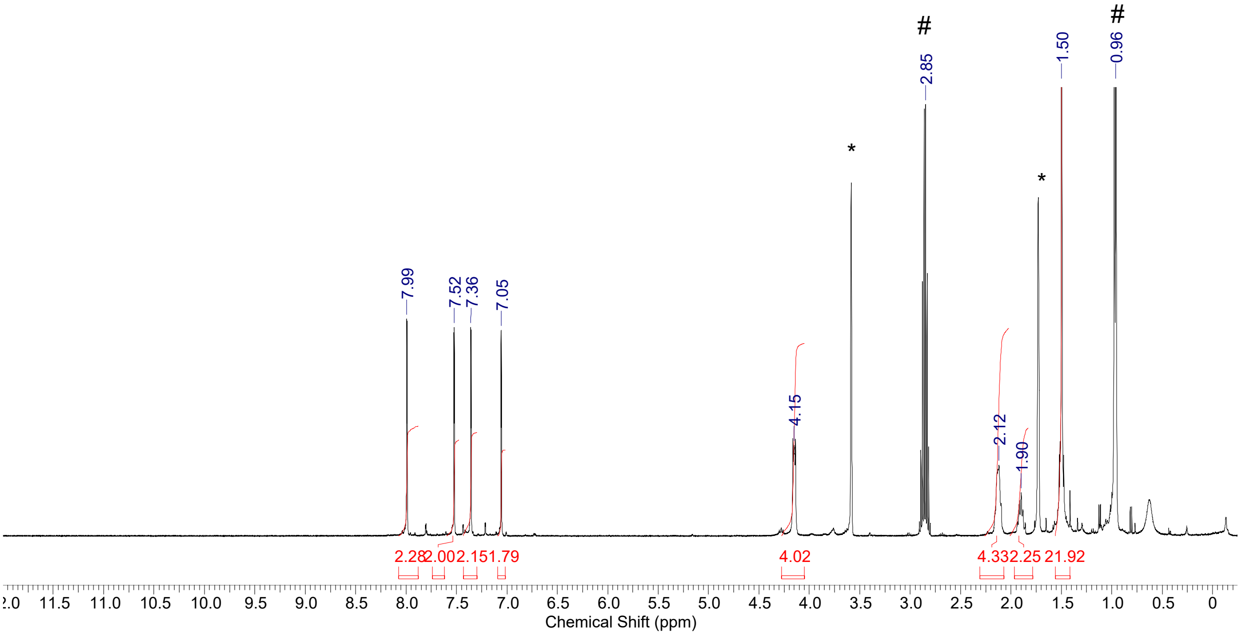

*Figure 4: ^{1}H NMR spectrum (400 MHz, THF-d$_8$(*)) of [Li(bimca^{C5})] (**2**); generated with LDA (#); HDA(#).*

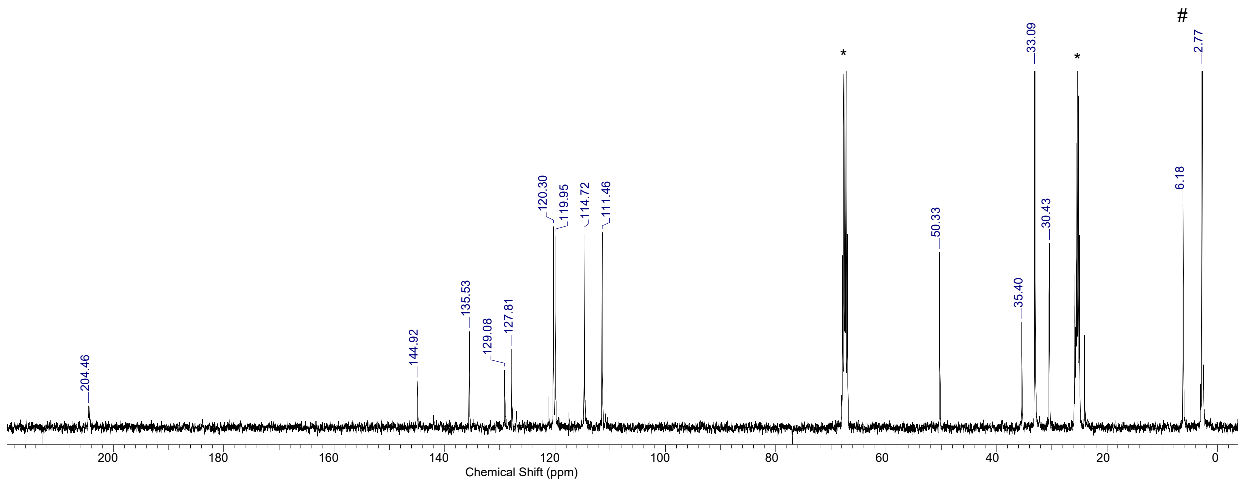

*Figure 5: ^{13}C NMR spectrum (100 MHz, THF-d_8 (*)) of [Li(bimca^{C5})] (**2**) generated with LiHMDS (#); HMDS (#).*

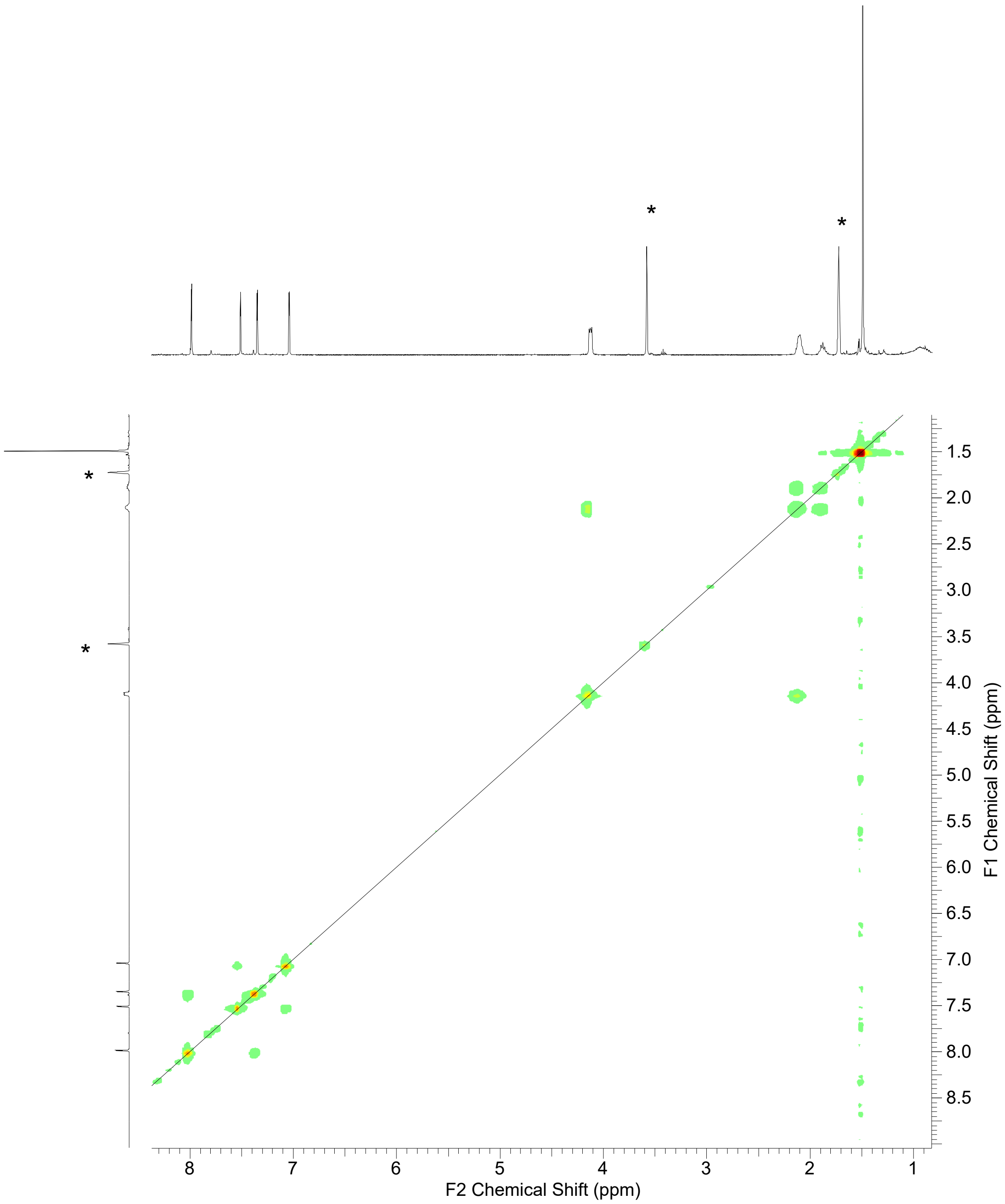

*Figure 6: ^{1}H,^{1}H COSY-NMR spectrum (detail) (400 MHz, THF-d_8 (*)) of [Li(bimca^{C5})] (**2**).*

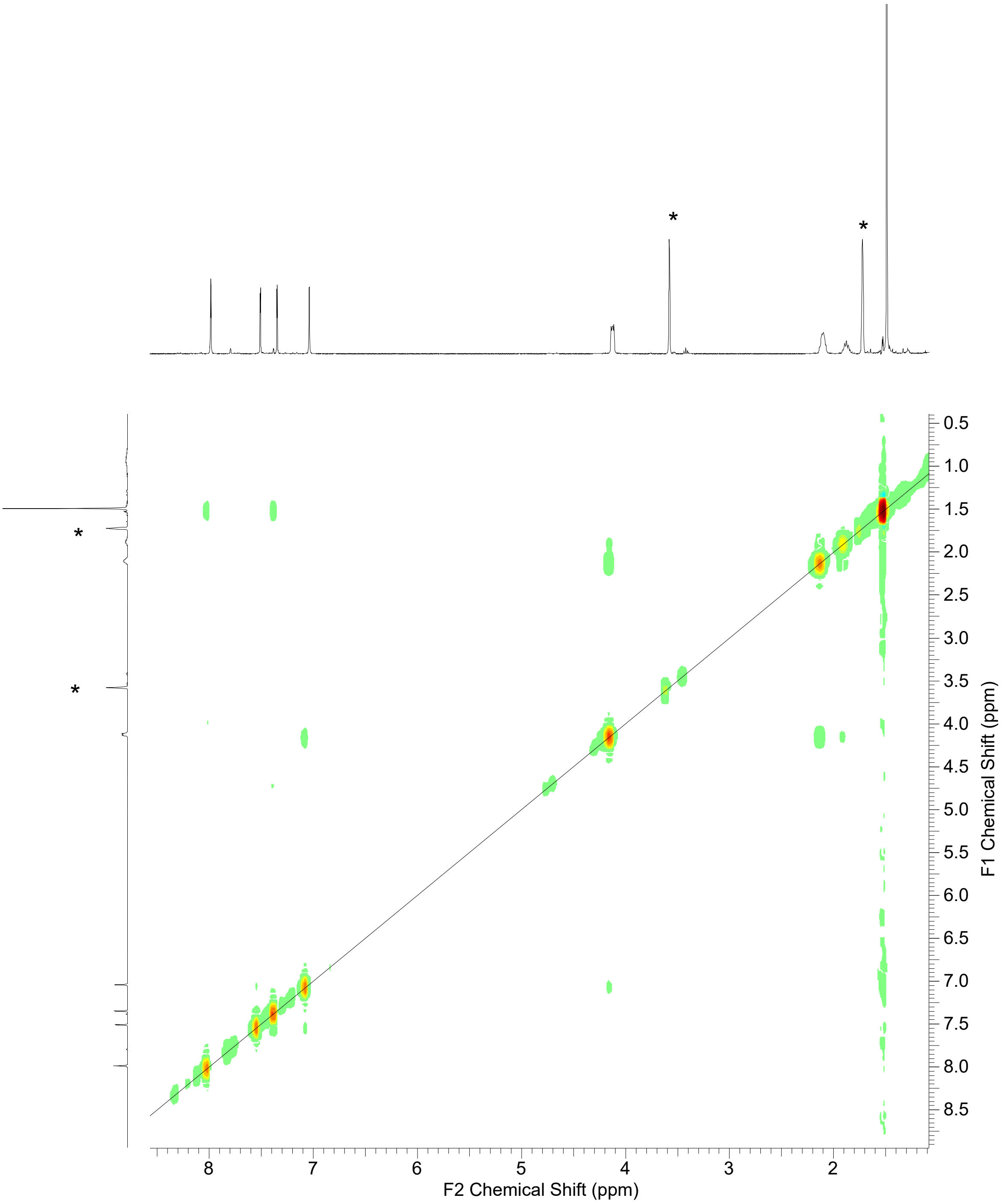

*Figure 7: ^{1}H,^{1}H NOESY-NMR spectrum (detail) (400 MHz, THF-d_8 (*)) of [Li(bimca^{C5})] (**2**).*

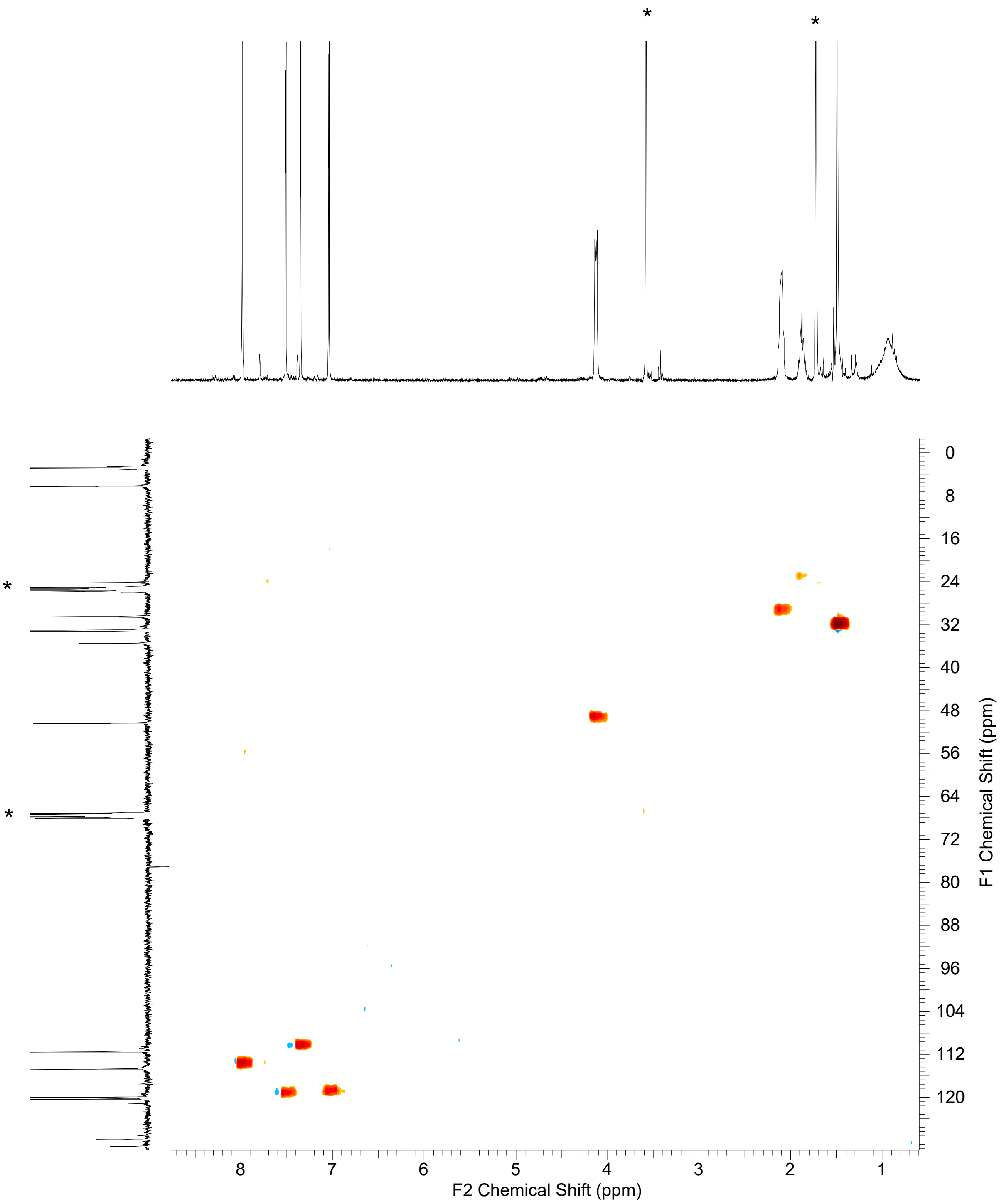

*Figure 8: $^{1}H,^{13}C$ HSQC-NMR spectrum (detail) (400 MHz, THF-d_8 (*)) of [Li(bimca^{C5})] (**2**).*

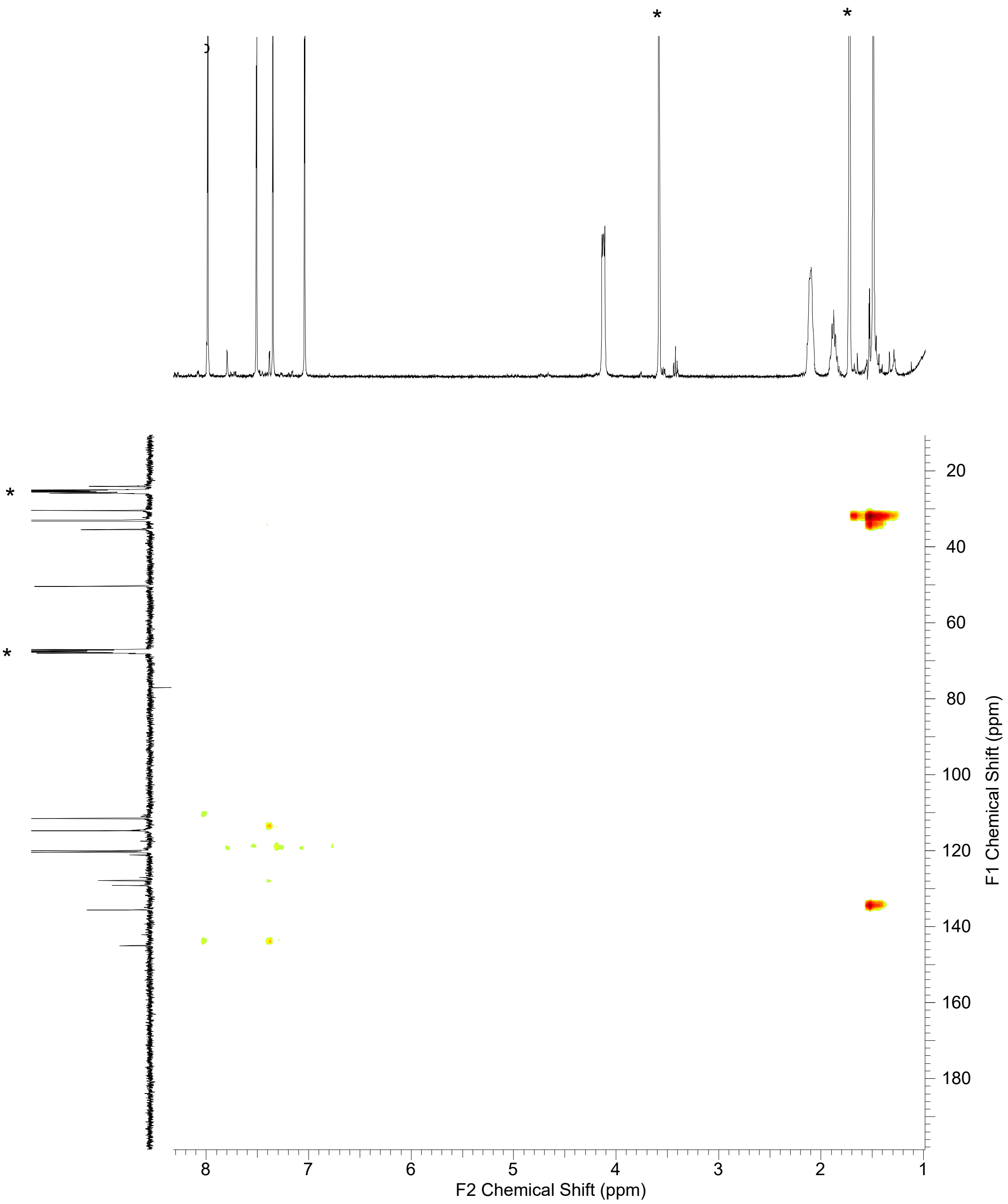

*Figure 9: ^{1}H,^{13}C HMBC-NMR spectrum (detail) (400 MHz, THF-d_8 (*)) of [Li(bimca^{C5})] (**2**).*

Low temperature measurement:

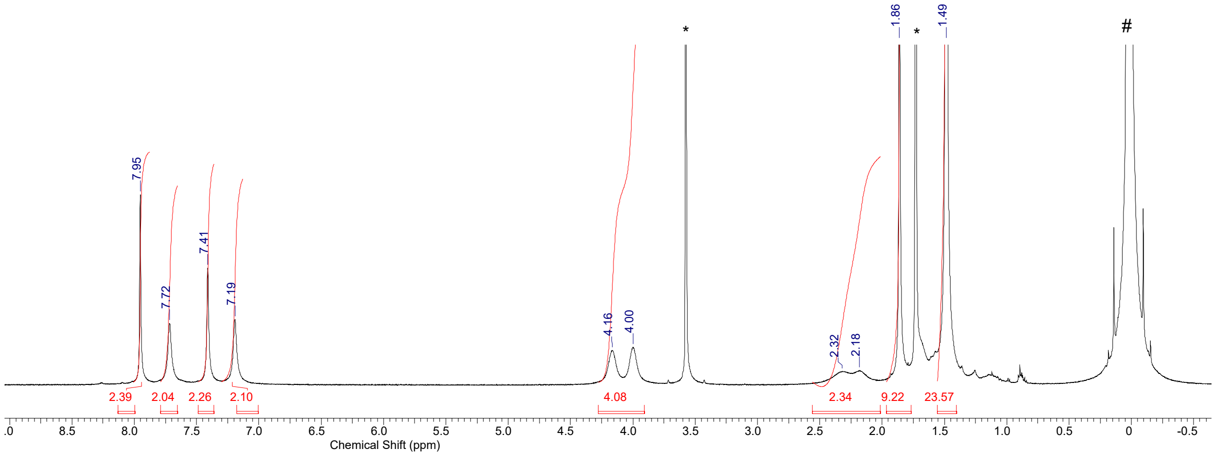

*Figure 10: ^{1}H NMR spectrum (500 MHz, THF-d$_8$ (*)) at -90 °C of [Li(bimca^{C5})] (**2**) generated with LiHMDS(#); HMDS (#).*

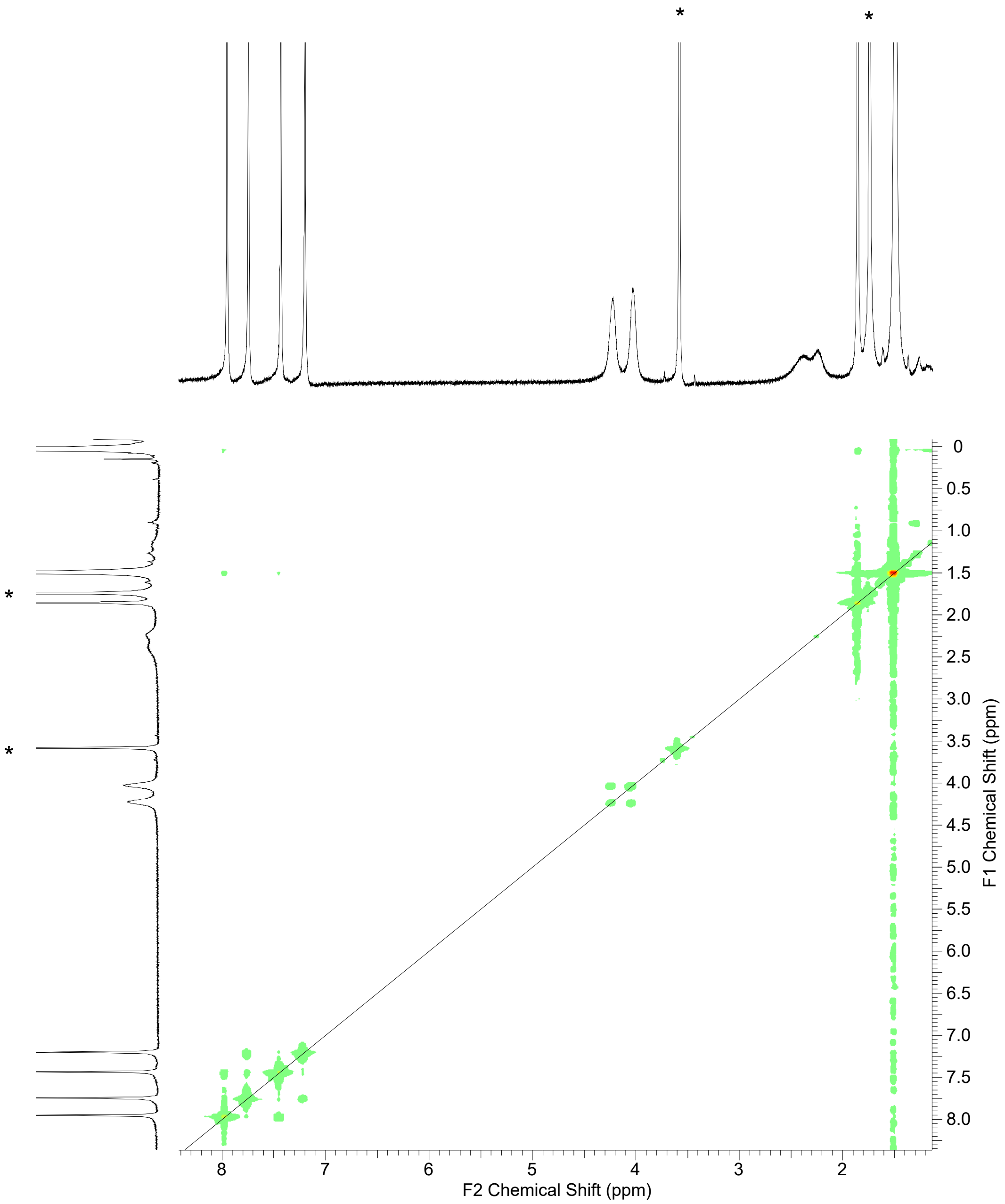

*Figure 11: ^{1}H, ^{1}H COSY spectrum (500 MHz, THF-d_8 (*)) (*)) at -90 °C of [Li(bimca^{C5})] (**2**).*

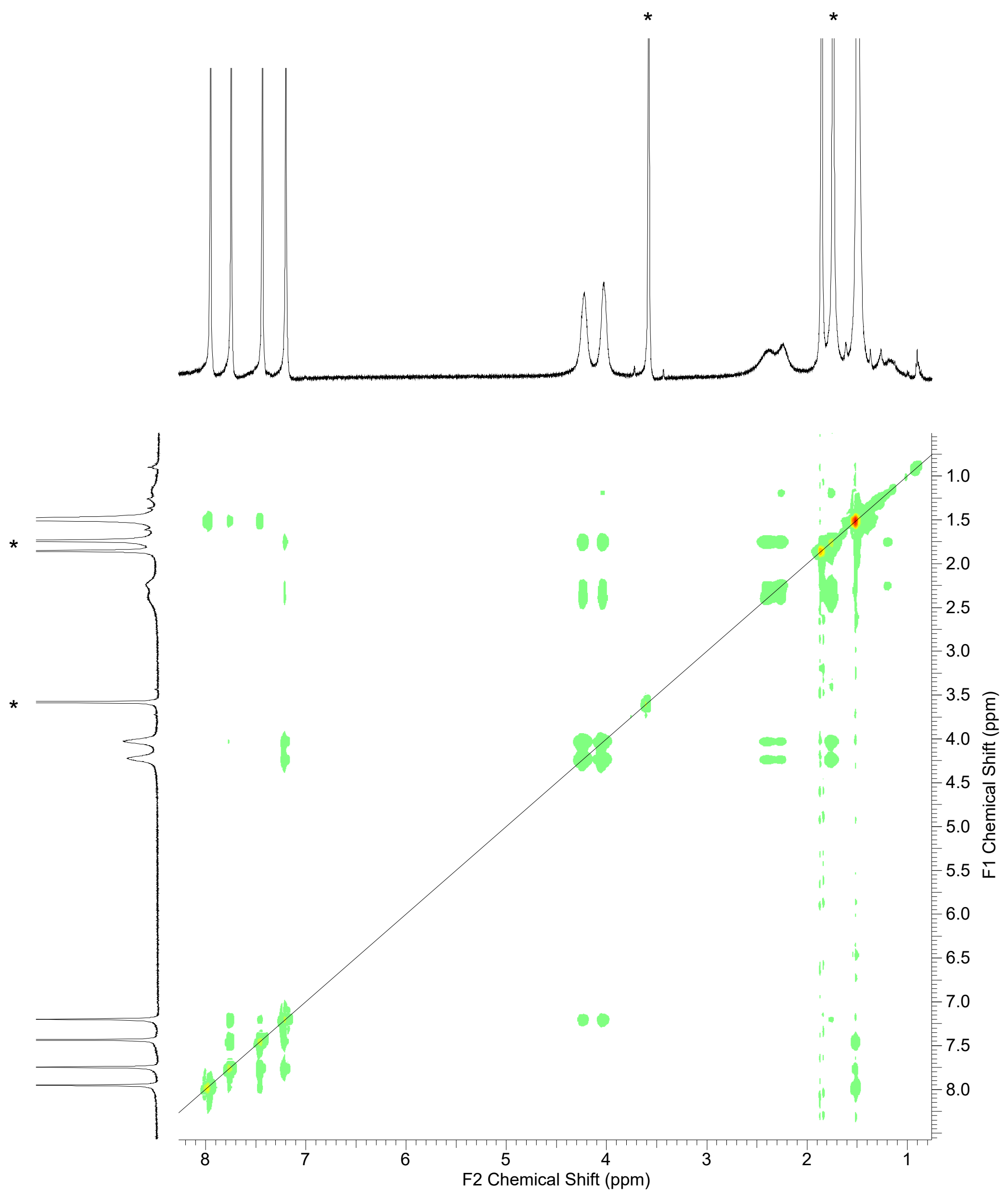

*Figure 12: ^{1}H, ^{1}H NOESY spectrum (500 MHz, THF-d8 (*)) at-90 °C of [Li(bimca^{C5})] (**2**).*

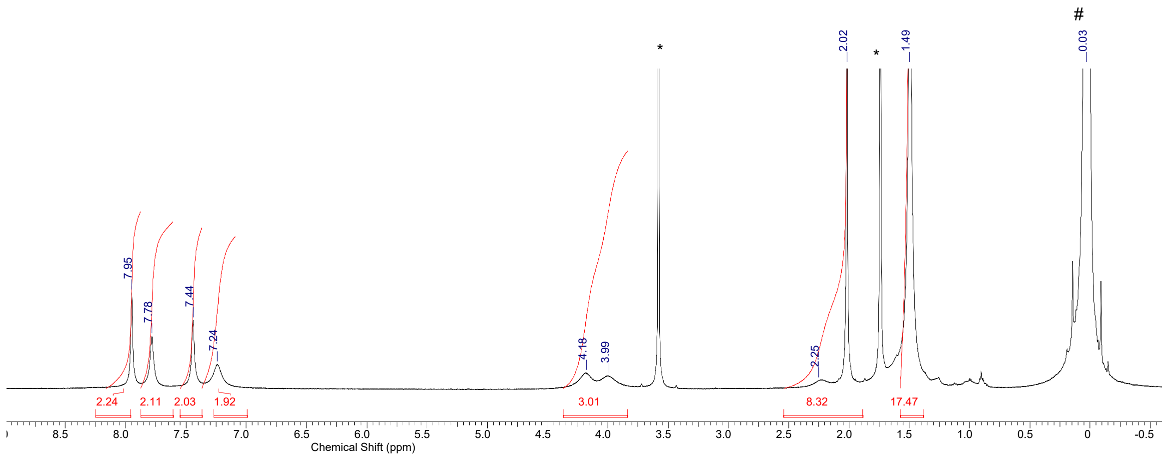

*Figure 13: ^{1}H NMR spectrum (500 MHz, THF-d$_8$ (*)) at -110 °C of [Li(bimca^{C5})] (**2**) generated with LiHMDS (#); HMDS (#).*

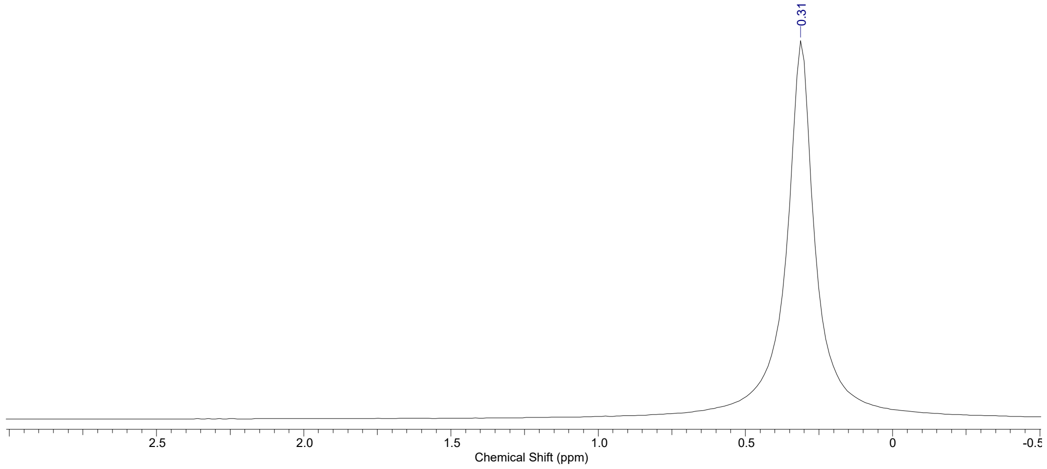

*Figure 14: ^{7}Li NMR spectrum (300 MHz, THF-d$_8$) of [Li(bimca^{C5})] (**2**) at RT.*

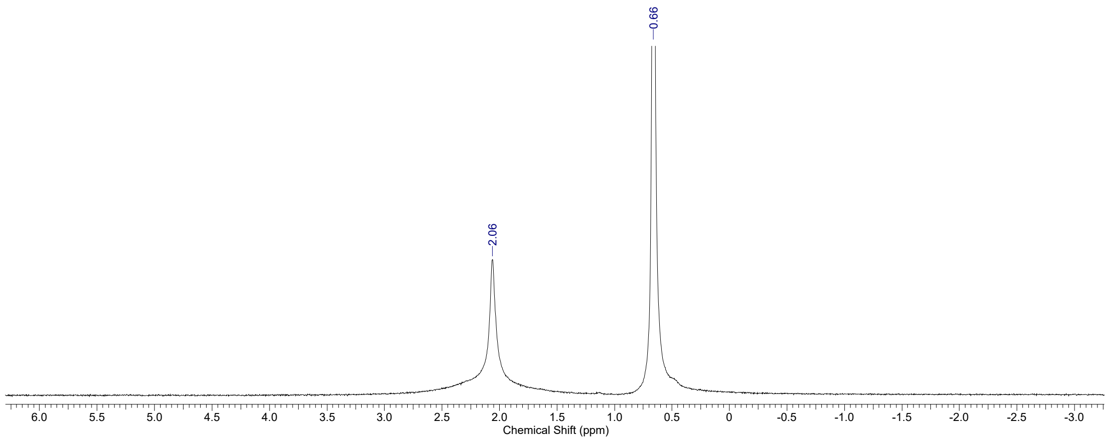

*Figure 15: ^{7}Li NMR spectrum (500 MHz, THF-d$_8$) at -90 °C of [Li(bimca^{C5})] (**2**) generated with LiHMDS(#); HMDS (#).*

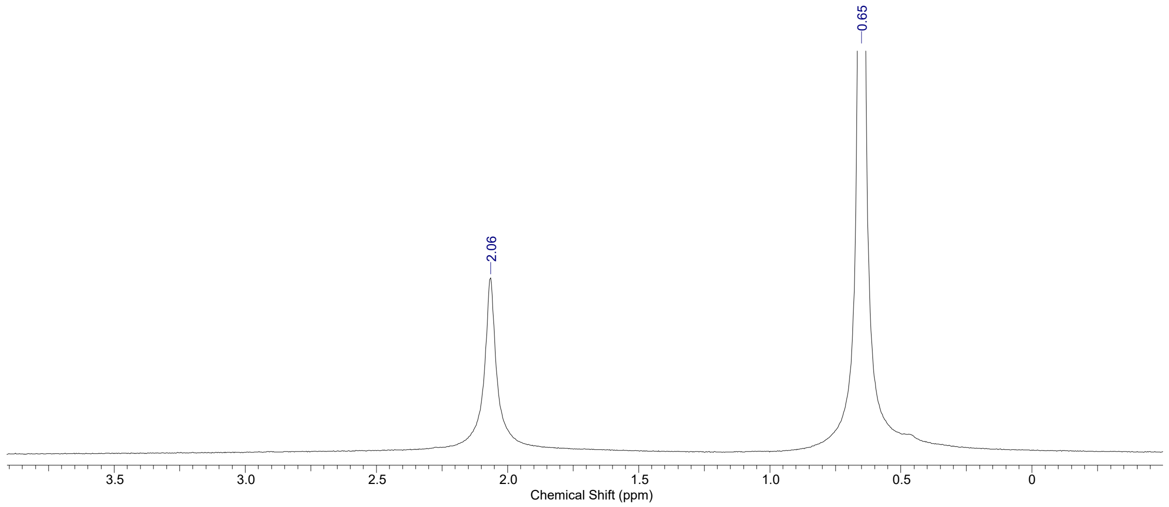

*Figure 16: ^{7}Li NMR spectrum (500 MHz, THF-d$_8$) at -110 °C of [Li(bimca^{C5})] (**2**) with LiHMDS(#); HMDS (#).*

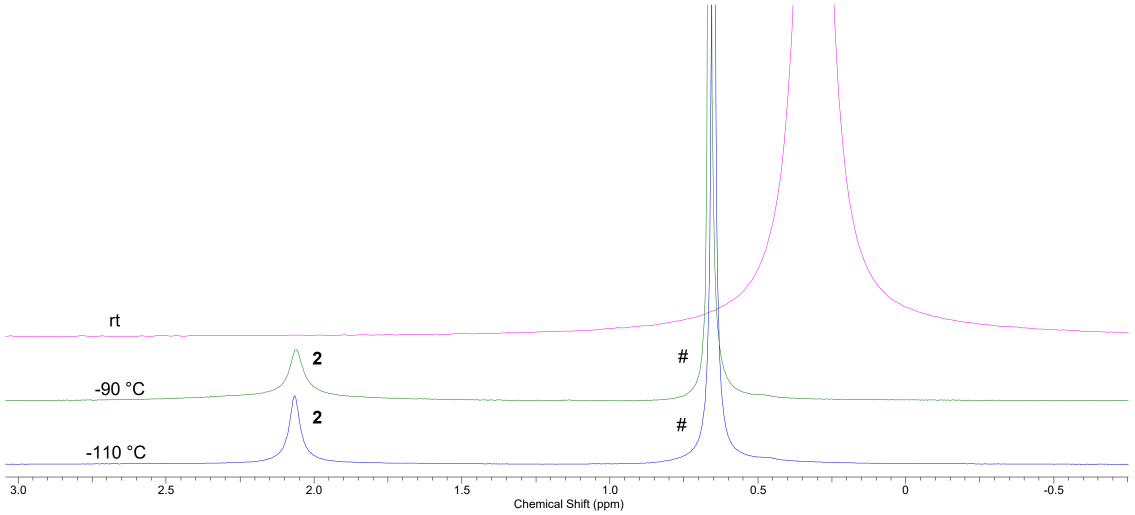

*Figure 17: VT ^{7}Li NMR spectra (300 and 500 MHz, THF-d_8) of [Li(bimca^{C5})] (**2**) generated with LiHMDS(#); LiX(#).*

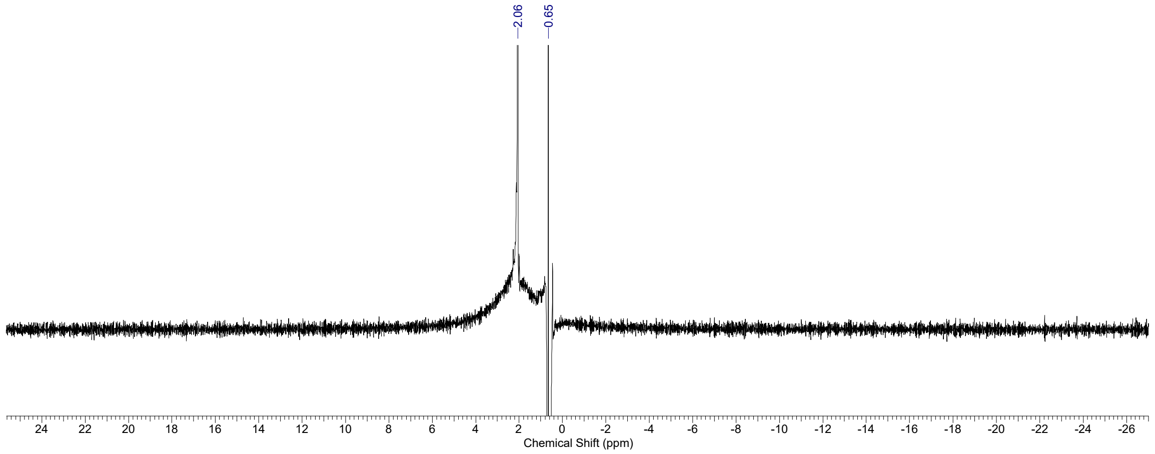

*Figure 18: ^{7}Li NMR spectrum (500 MHz, THF-d_8) at -110 °C of [Li(bimca^{C5})] (**2**) generated with LiHMDS/HMDS (#). Resolution enhanced version of Figure 16~~Figure 16~~ by gaussian windows multiplication (apodisation).*

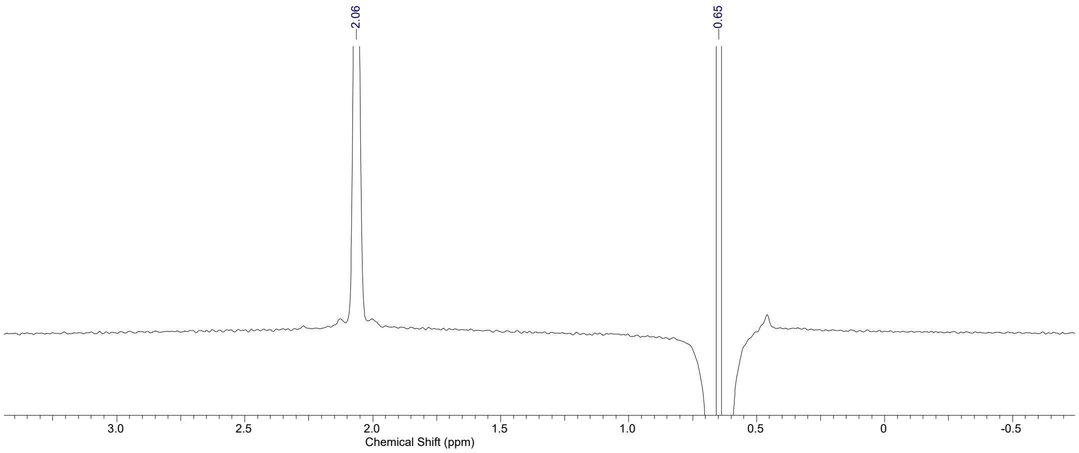

Figure 19: Detail of Figure 18~~Figure 18~~ to emphasize the ^{13}C satellites ($^{1}J(^{7}Li^{13}C)$) of the signal at 2.06 ppm.

[K(bimca^{C5})] (3):

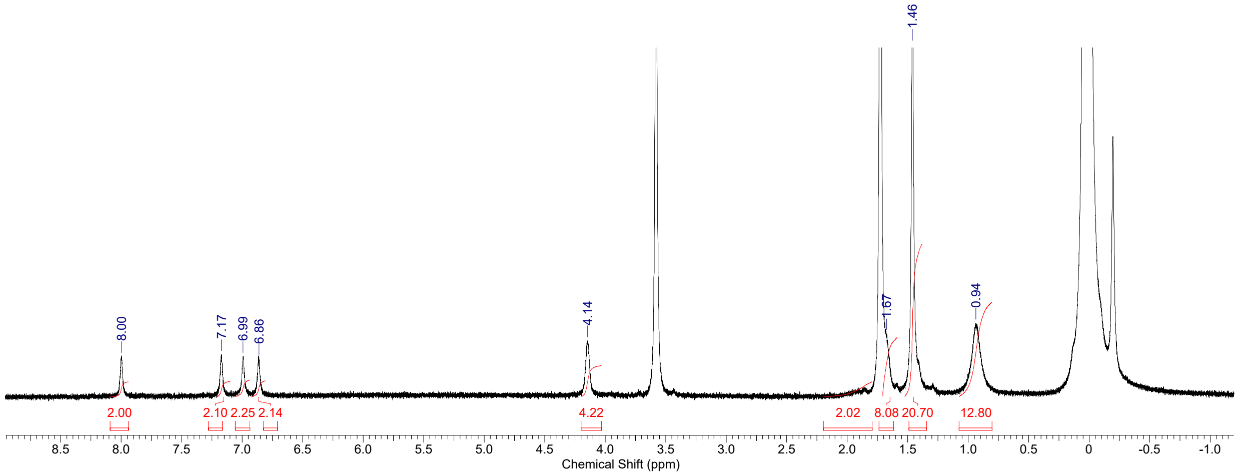

*Figure 20: ^{1}H NMR spectrum (400 MHz, THF-d$_8$ (*)) of [K(bimca^{C5})] (**3**) generated with KHMDS(#); HMDS (#).*

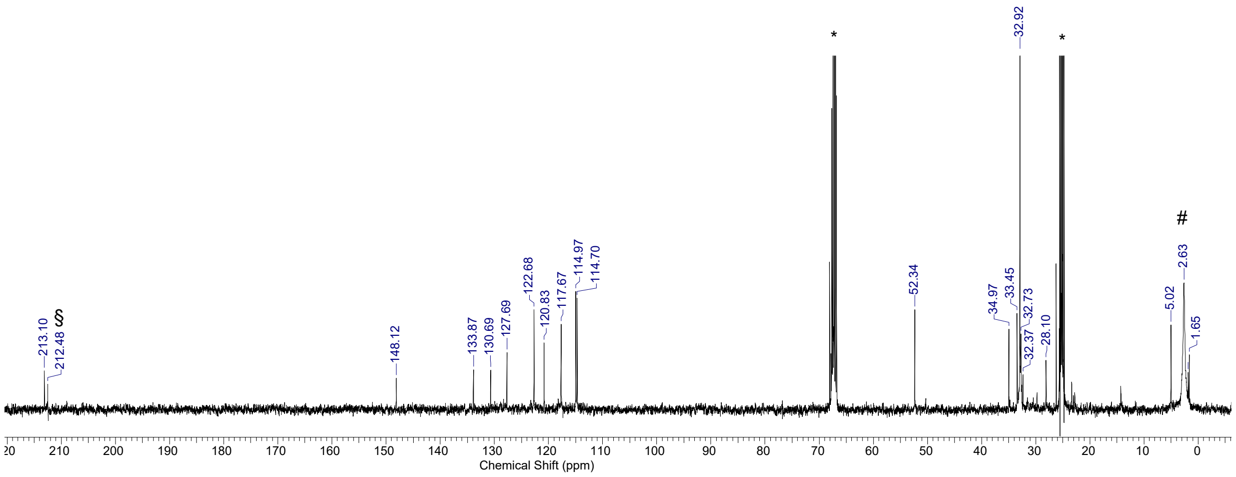

*Figure 21: ^{13}C NMR spectrum (100 MHz, THF-d$_8$ (*)) of [K(bimca^{C5})] (**3**) generated with KHMDS(#); HMDS (#), artefact (§).*

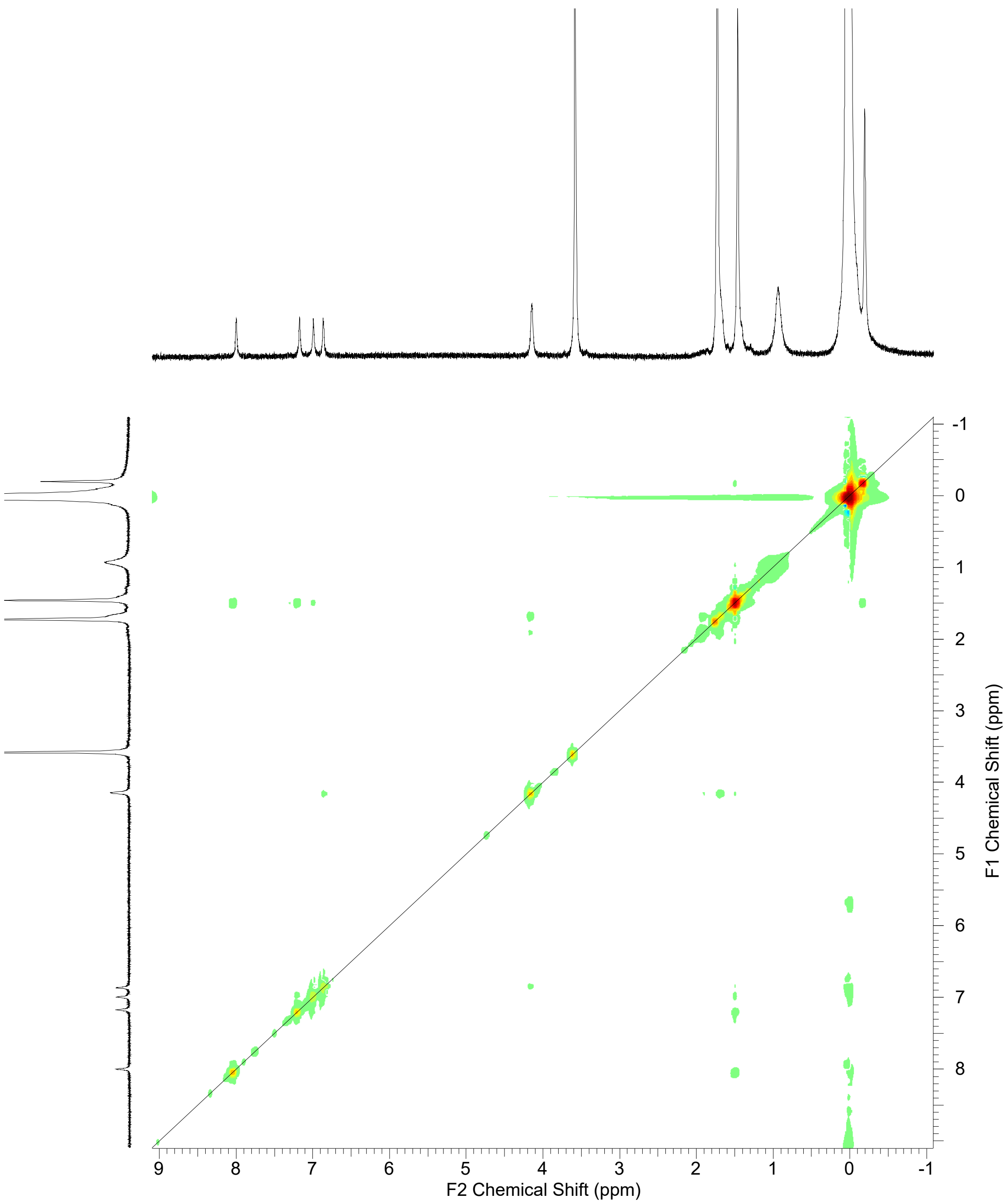

*Figure 22: $^{1}H,^{1}H$ NOESY-NMR spectrum (500 MHz, THF-d_8 (*)) of [K(bimca^{C5})] (**3**) (# = HMDS/KHMDS).*

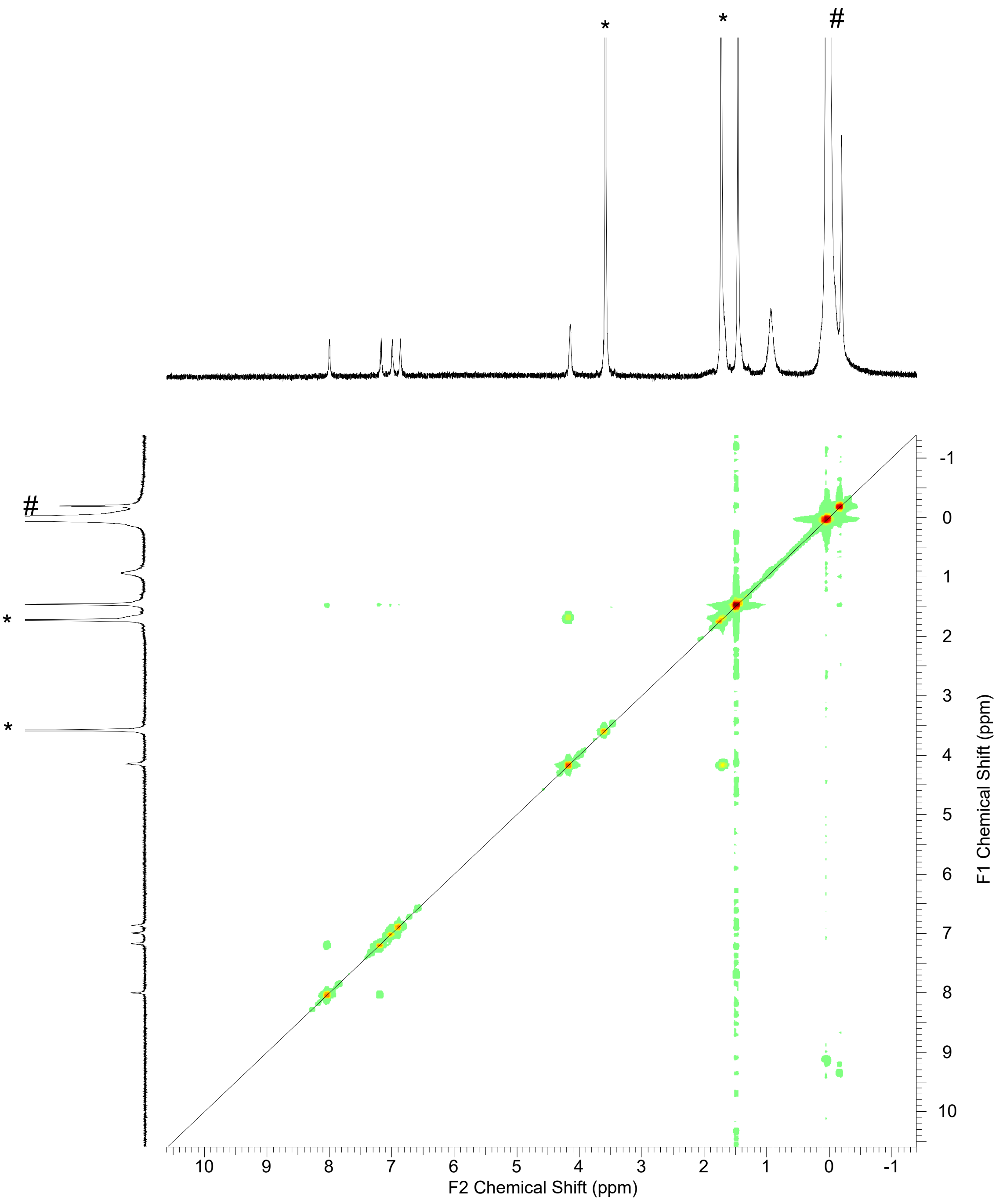

Figure 23: ^{1}H,^{1}H COSY-NMR spectrum (500 MHz, THF-d$_8$ *(*)) of [K(bimca*^{C5}*)]* ***(3)*** *(# = HMDS/KHMDS.)*

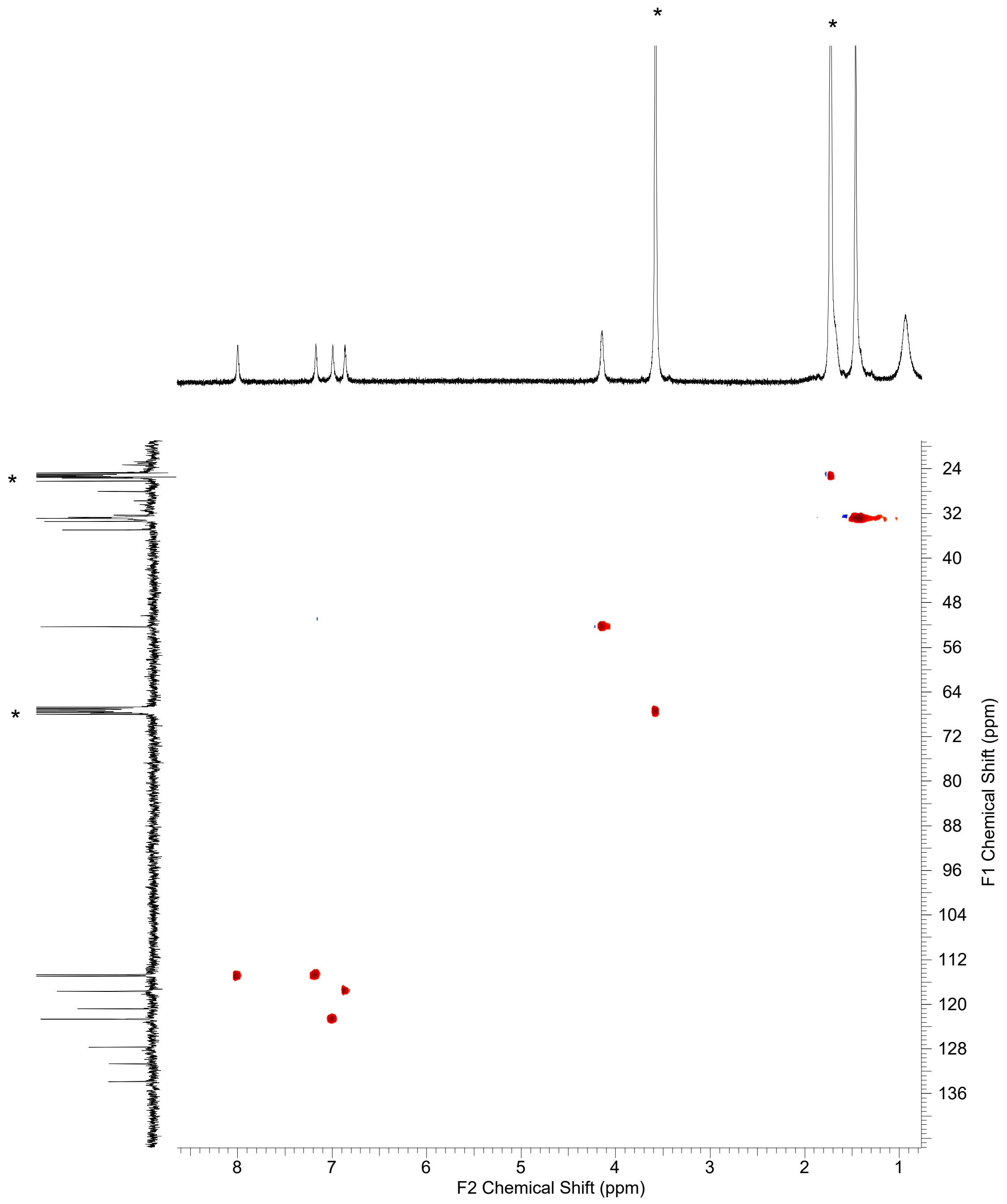

*Figure 24: ^{1}H,[13]H HSQC NMR spectrum (500 MHz, THF-d8 (*)) of [K(bimca^{C5})] (**3**).*

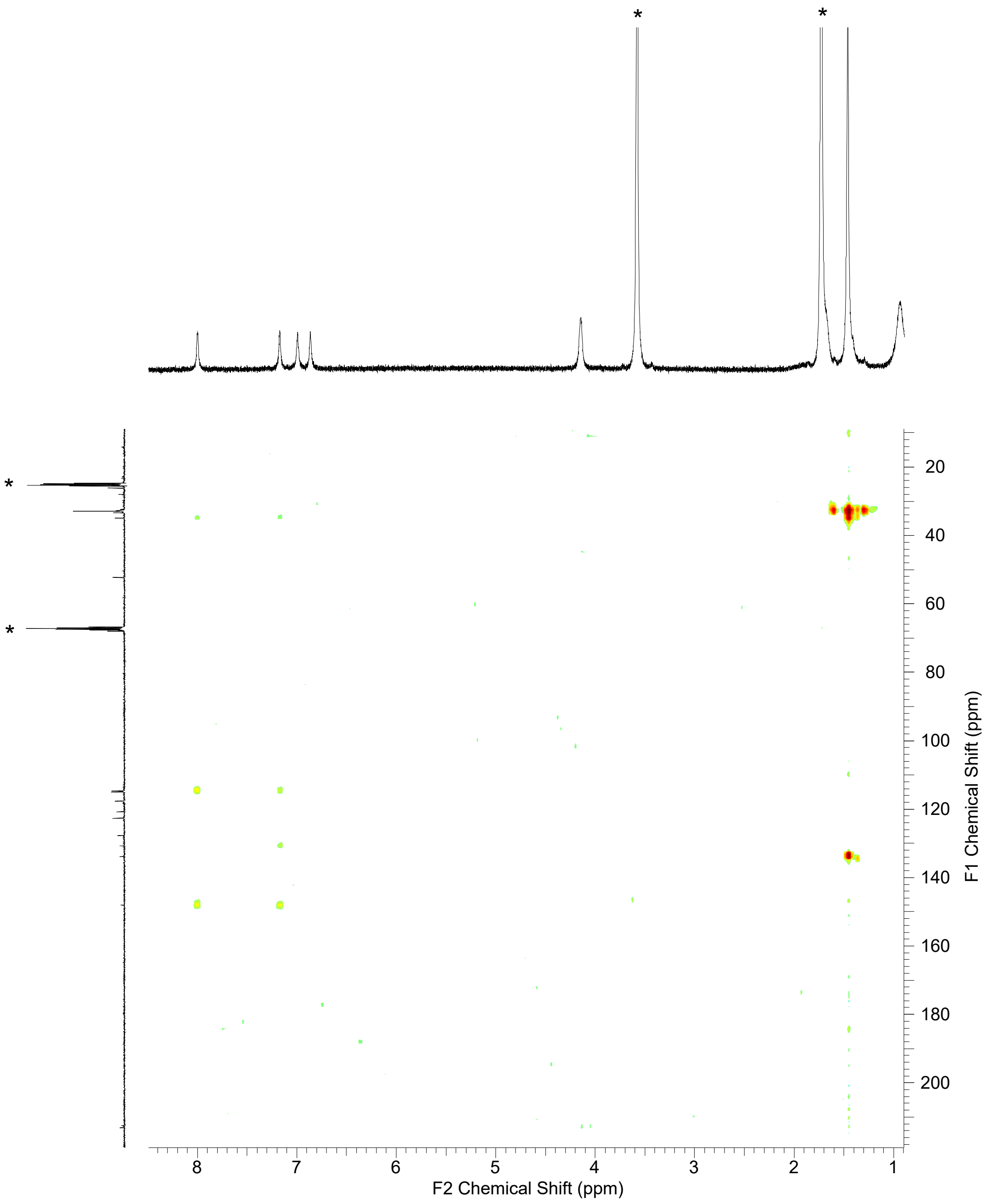

*Figure 25: ^{1}H,^{13}C HMBC NMR spectrum (400 MHz, THF-d_8 (*)) of [K(bimca^{C5})] (**3**).*

[Ru(bimca^{C5})Cp*] (4):

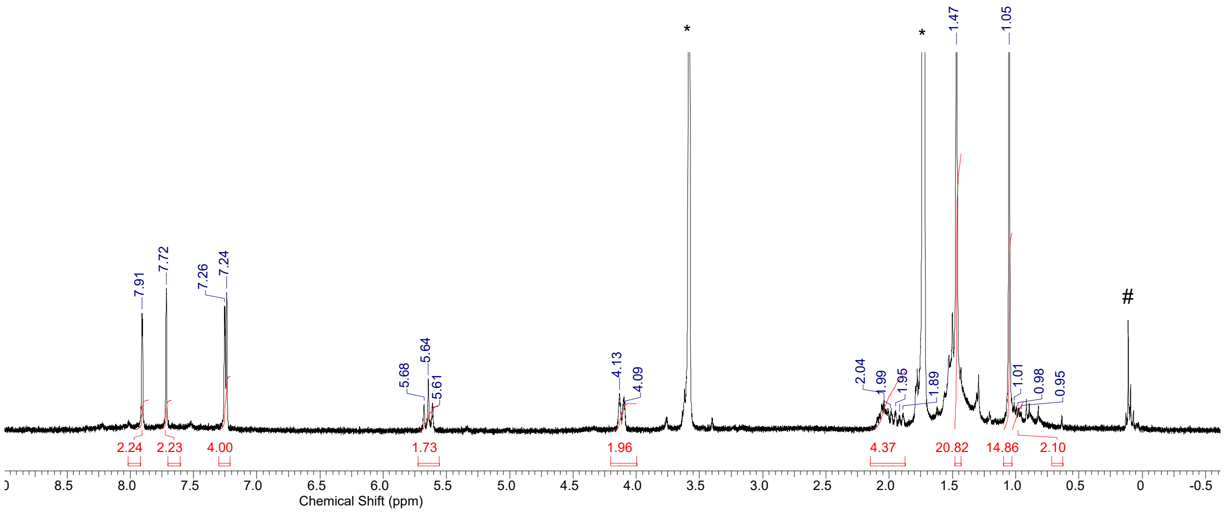

Figure 26: ^{1}H NMR spectrum (400 MHz, THF-d$_8$ (*)) of [Ru(bimca^{C5})Cp] (**3**) HMDS/grease (#).*

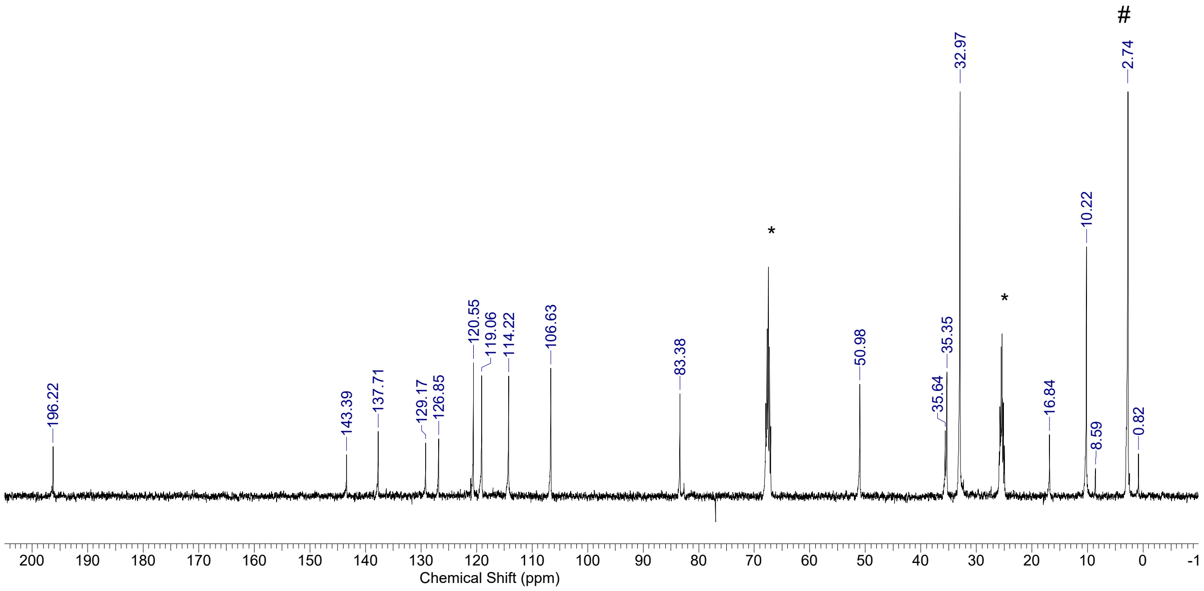

Figure 27: ^{13}C NMR spectrum (100 MHz, THF-d$_8$ (*)) of [Ru(bimca^{C5})Cp] (**4**); HMDS (#).*

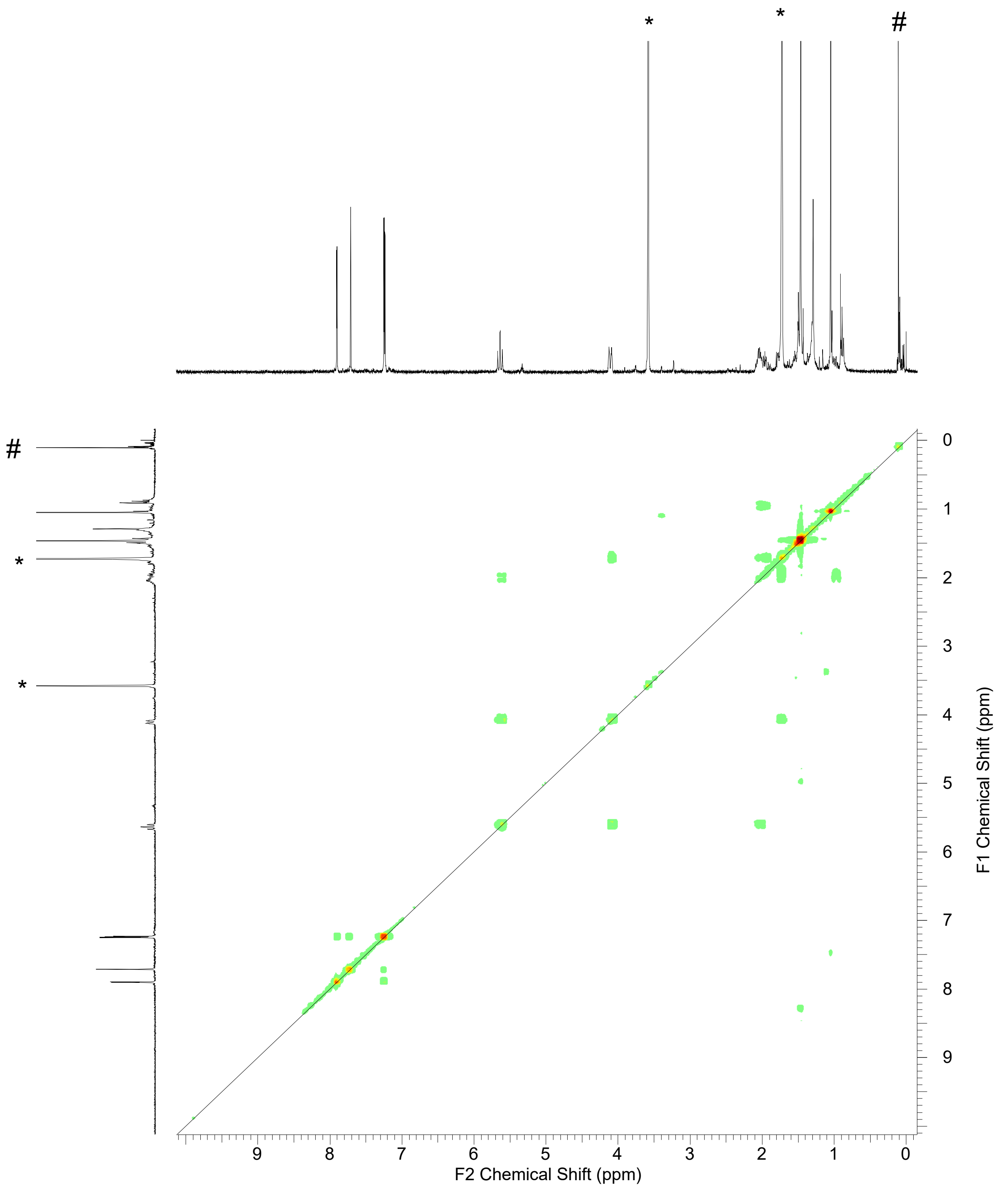

Figure 28: ^{1}H^{1}H COSY NMR spectrum (THF-d_8 (*)) of [Ru(bimca^{C5})Cp] (**4**); HMDS (#).*

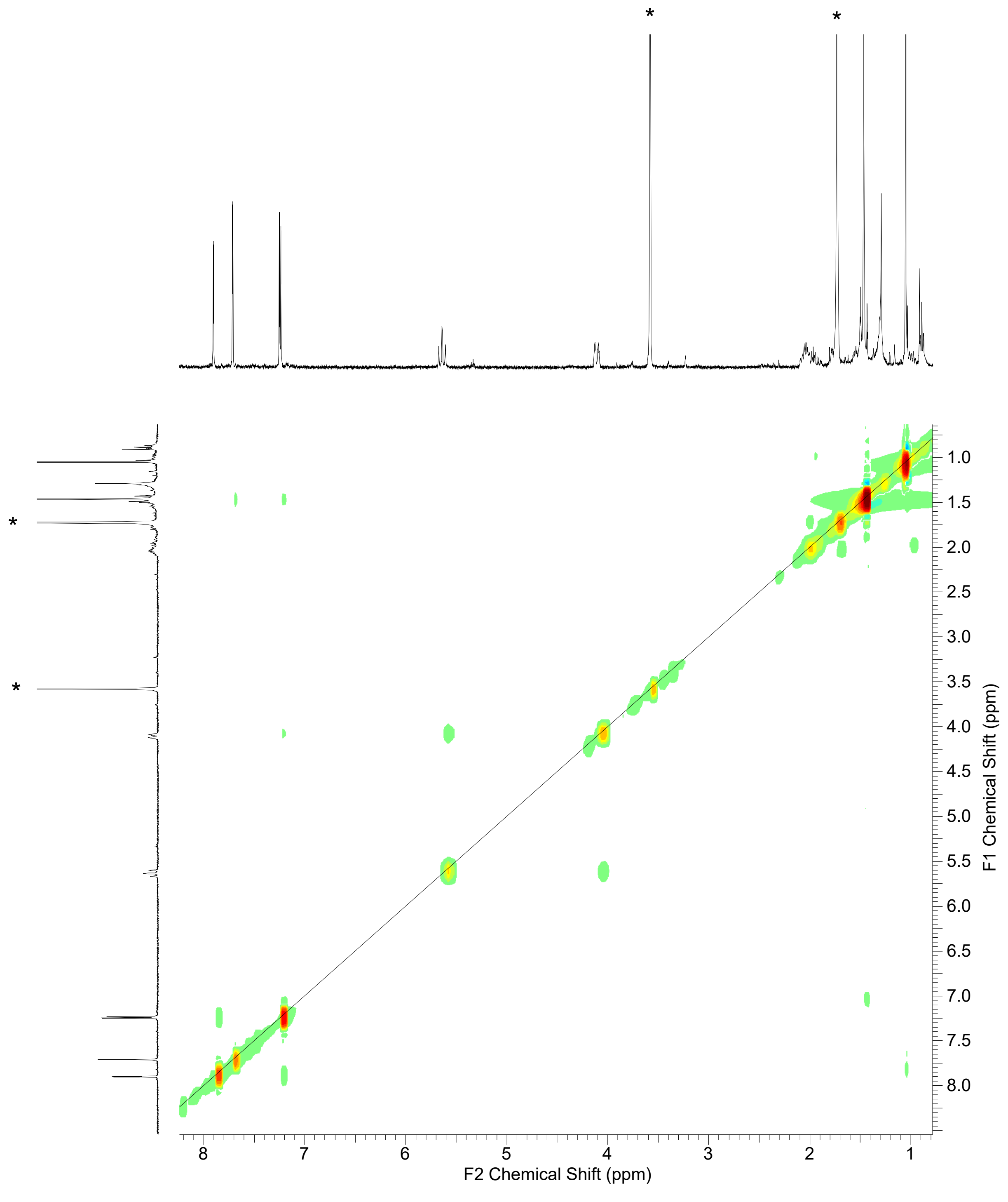

Figure 29: $^1H^1H$ COSY NMR spectrum (detail) (THF-d_8 (*)) of [Ru(bimca^{C5})Cp] (**4**).*

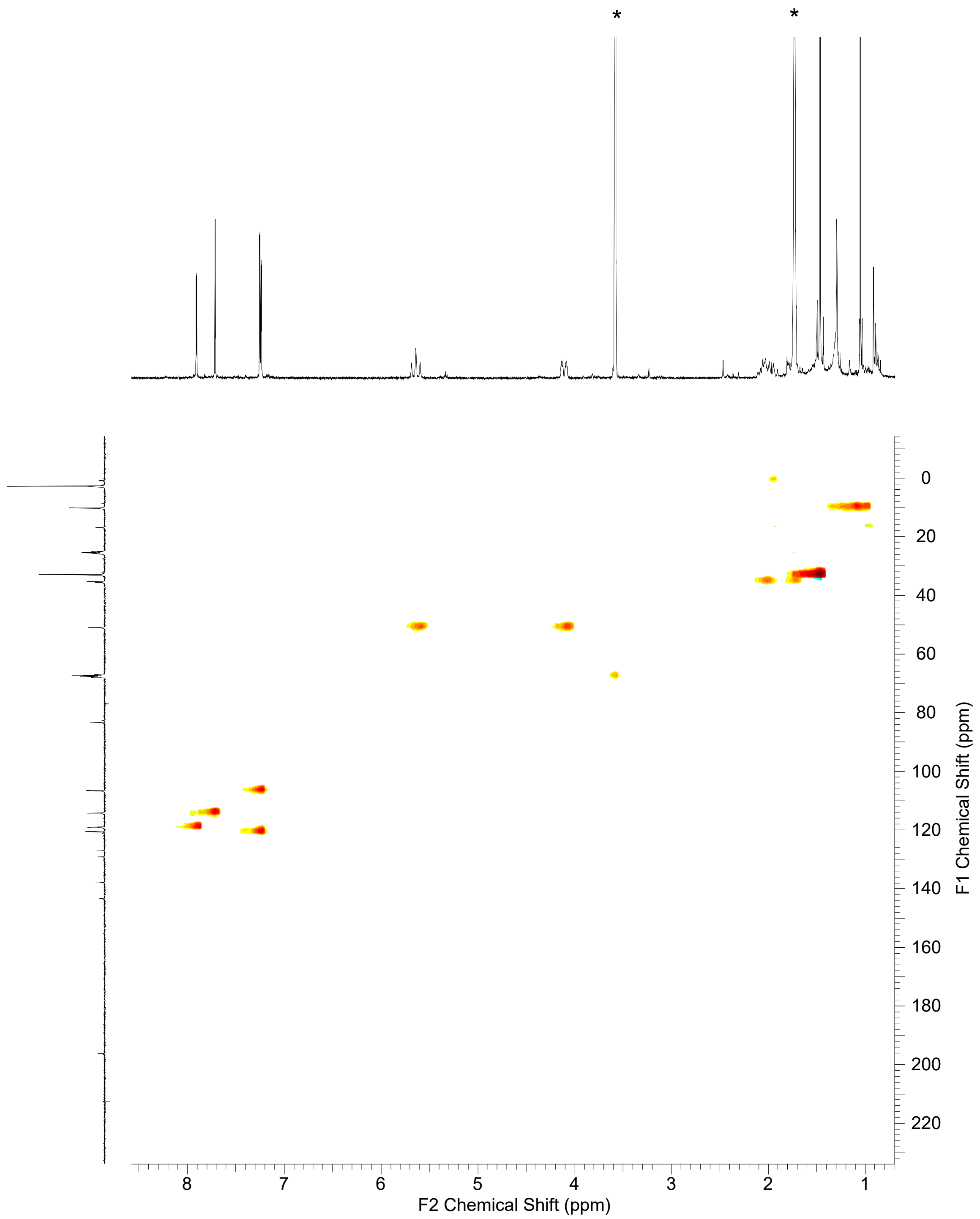

Figure 30: $^{1}H,^{13}C$ HSQC NMR spectrum (detail) (THF-d_8 (*)) of [Ru(bimca^{C5})Cp] (**4**).*

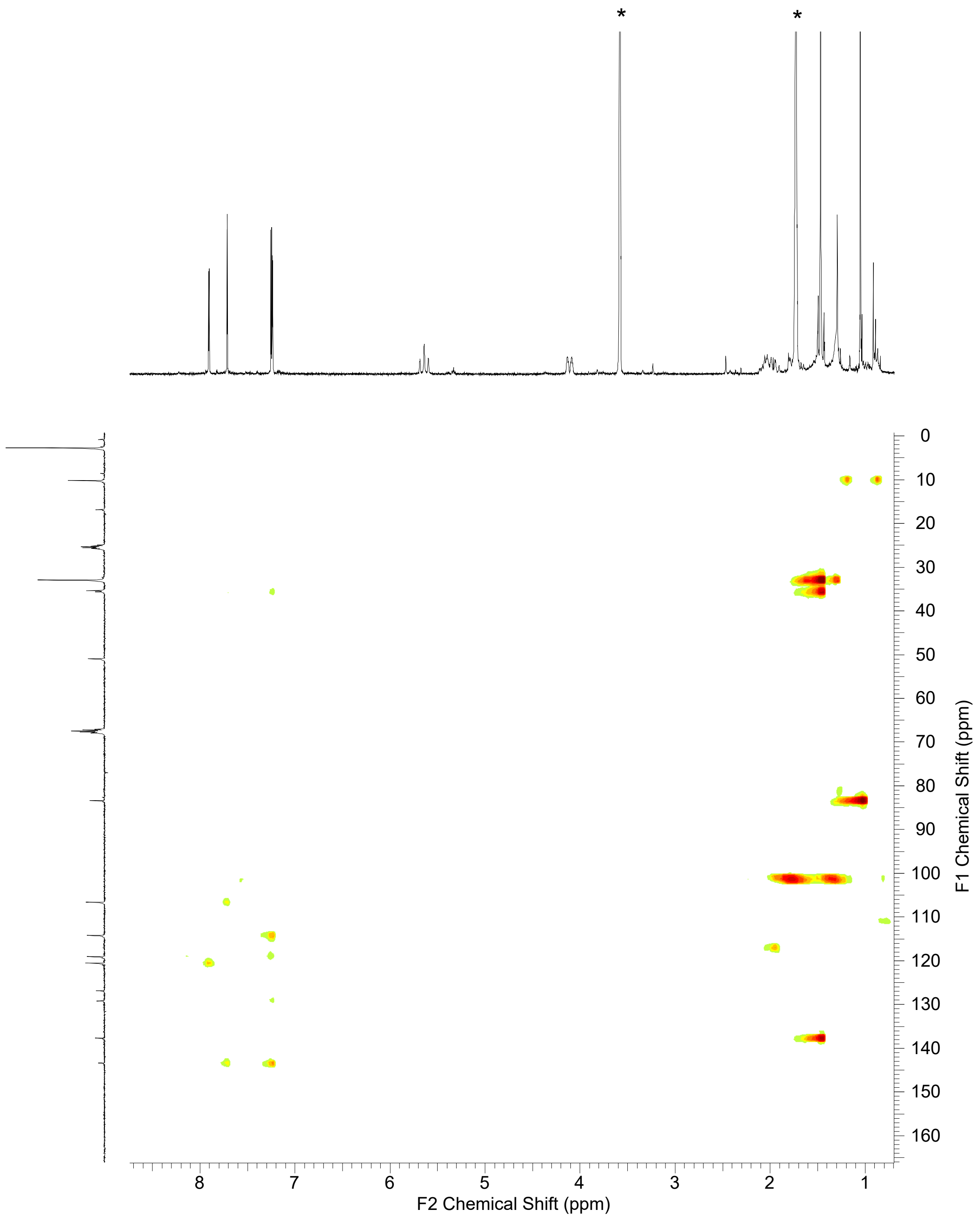

Figure 31: ^{1}H,^{13}C HMBC NMR spectrum (detail) (THF-d$_8$ (*)) of [Ru(bimca^{C5})Cp] (**4**).*

[Ru(bimca^{C5H8})Br(PPh$_3$)] (5):

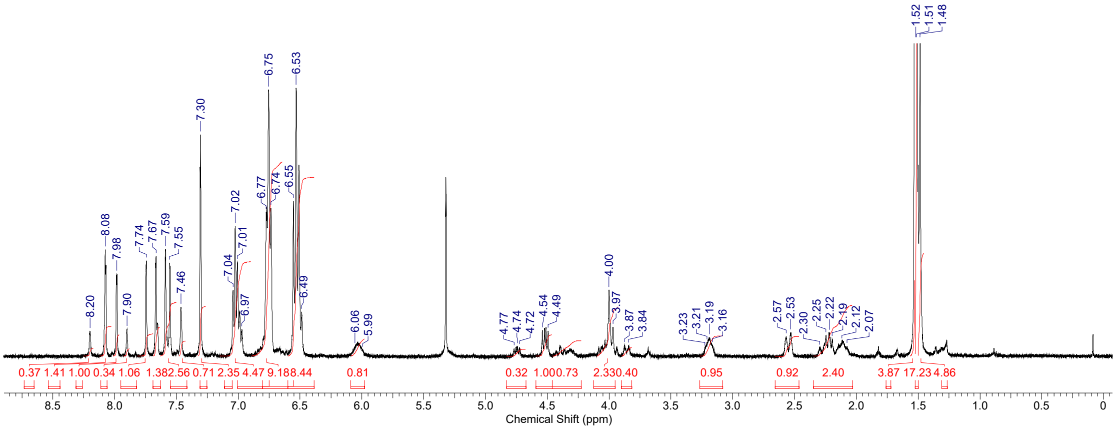

*Figure 32: ^{1}H NMR spectrum (400 MHz, DCM-d$_2$ (*)) of [Ru(bimca^{C5H8})Br(PPh$_3$)] (**5**).*

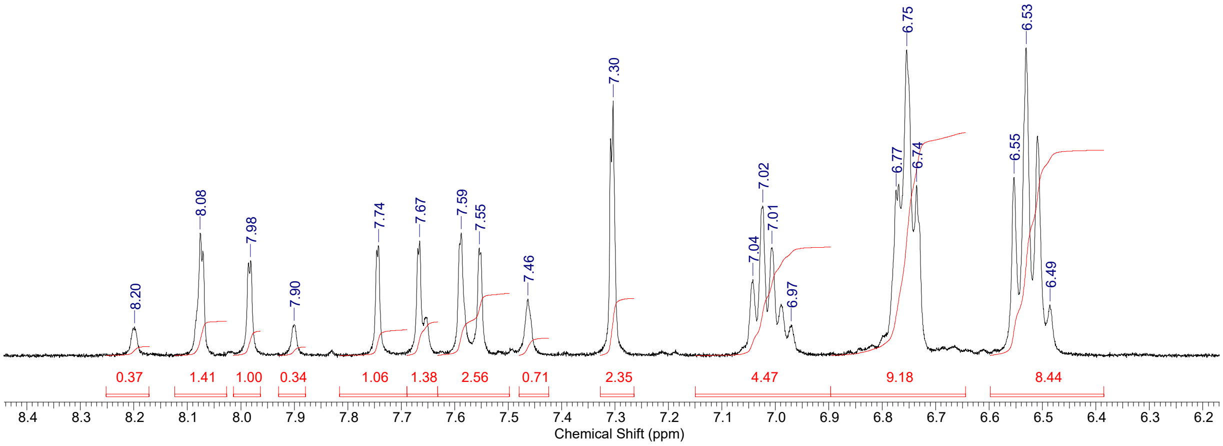

*Figure 33: ^{1}H NMR spectrum (detail) (400 MHz, DCM-d$_2$ (*)) of [Ru(bimca^{C5H8})Br(PPh$_3$)] (**5**).*

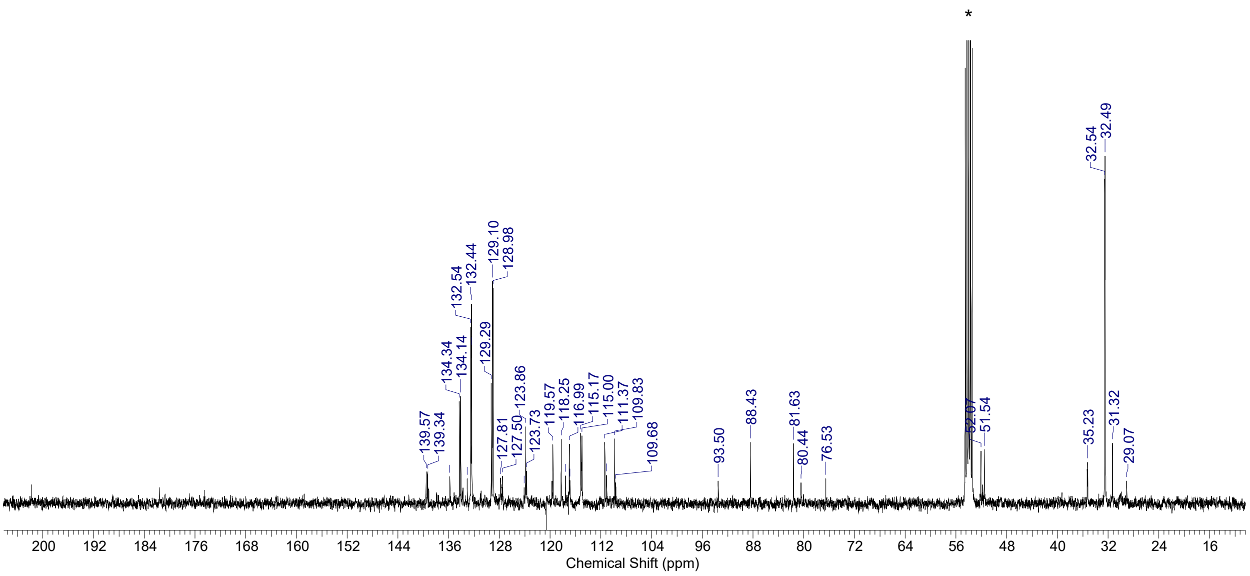

*Figure 34: ^{13}C NMR spectrum (100 MHz, DCM-d_2 (*)) of [Ru(bimca^{C5H8})Br(PPh_3)] (**5**).*

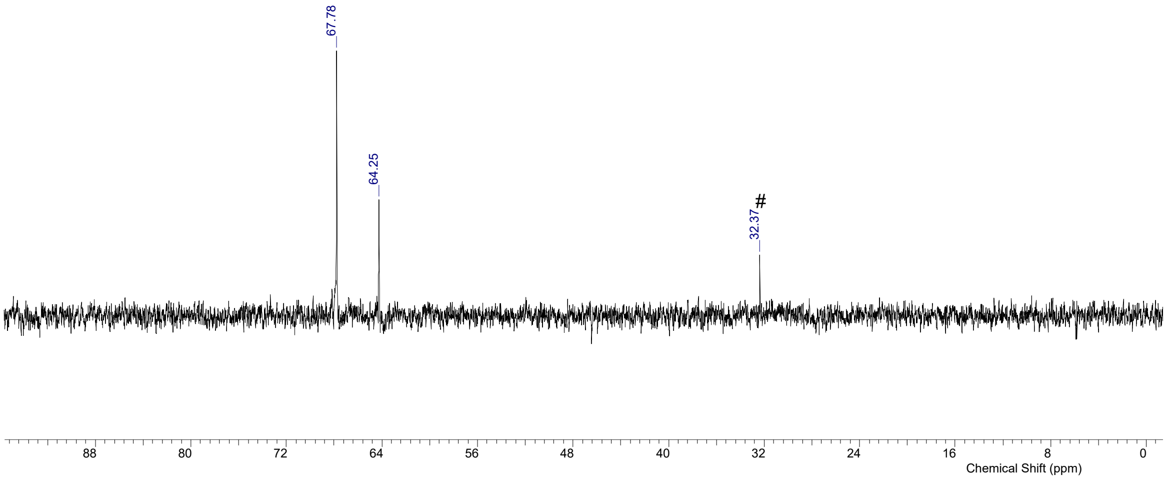

*Figure 35: ^{31}P NMR spectrum of [Ru(bimca^{C5H8})Br(PPh_3)] (**5**) in CD_3OD; triphenylphosphine oxide (#) .*

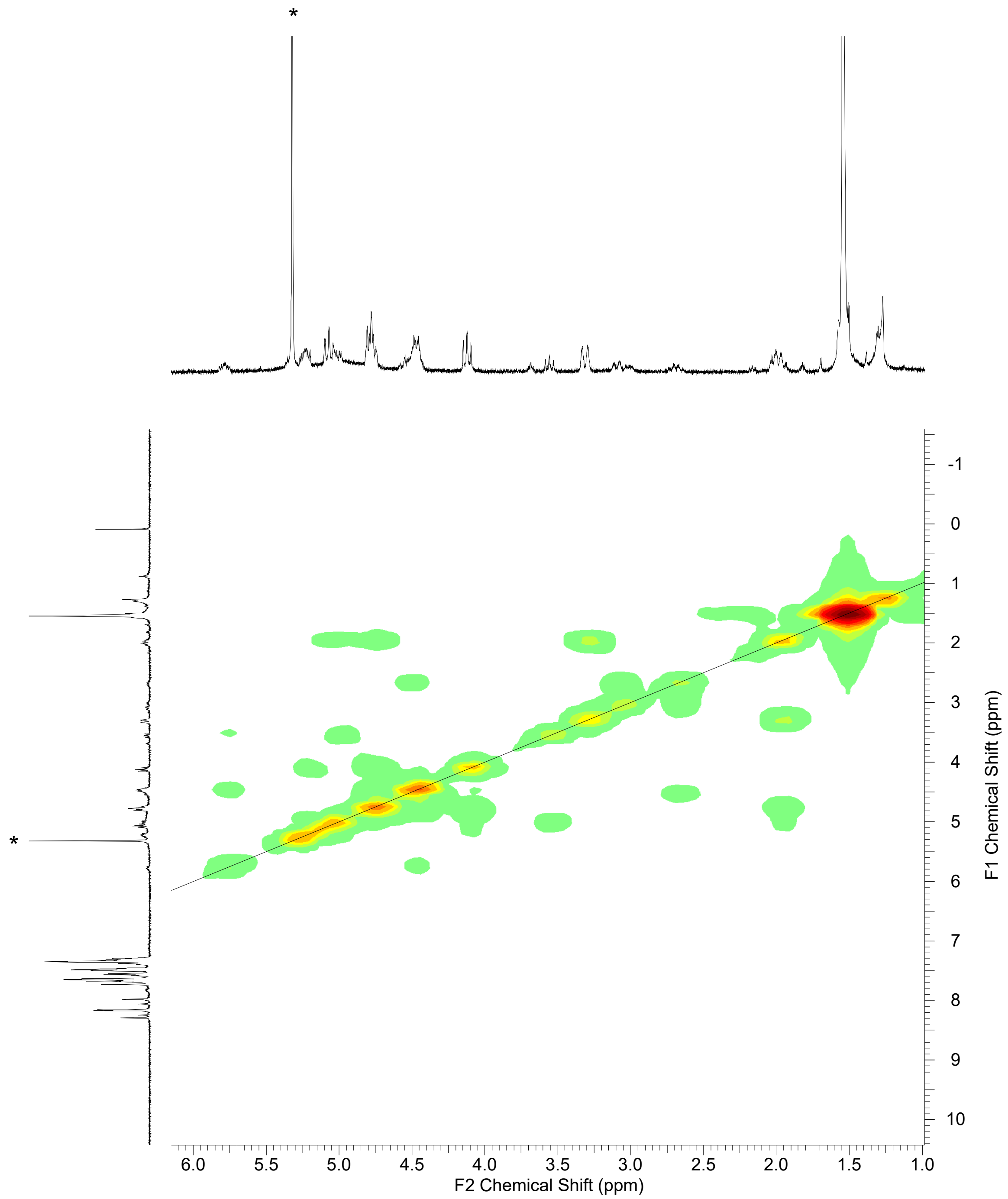

*Figure 36: ¹H,¹H COSY NMR spectrum (detail) (DCM-d_2 (*)) of [Ru(bimca^{C5H8})Br(PPh_3)] (**5**).*

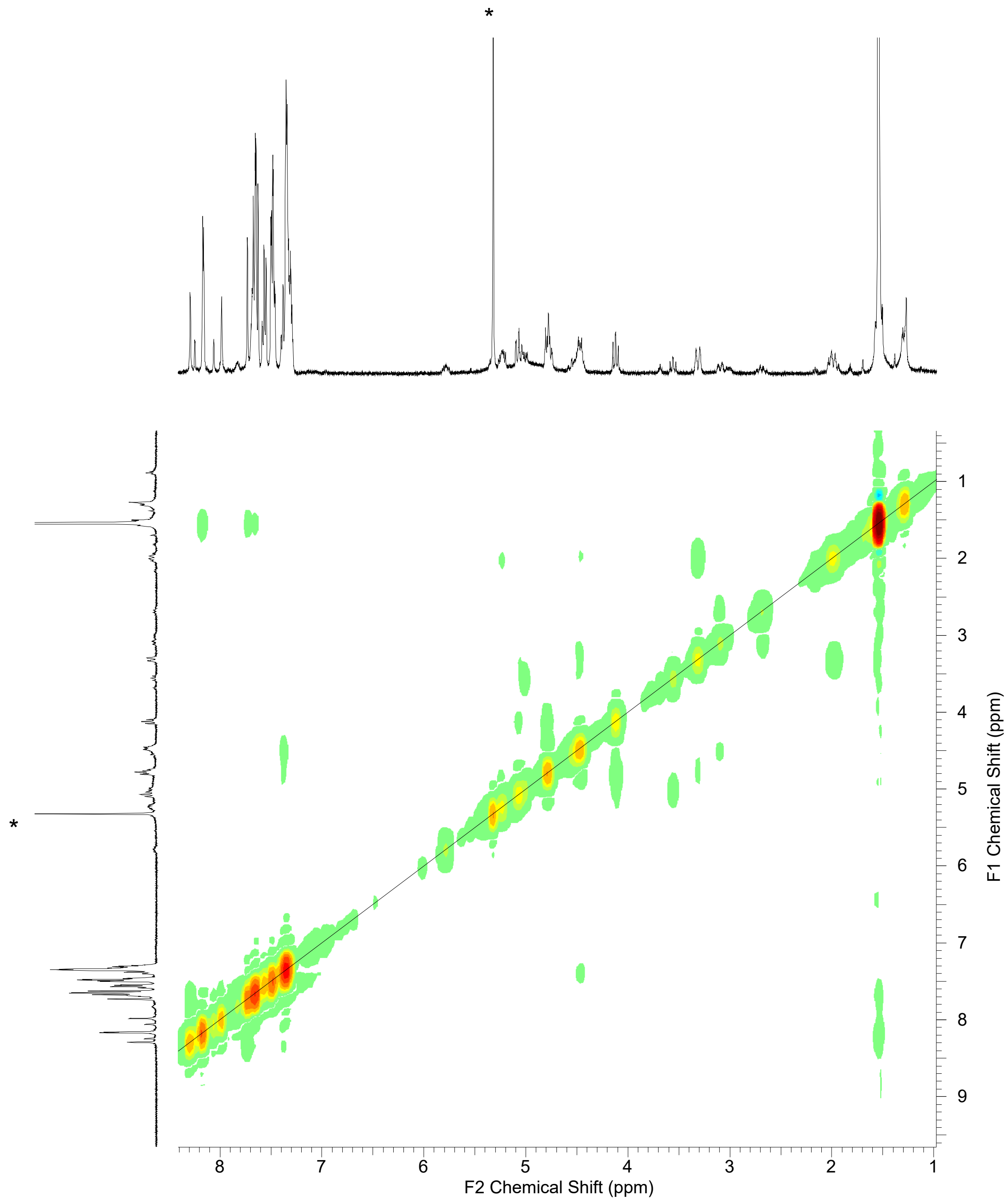

*Figure 37: ^{1}H,^{1}H NOESY NMR spectrum (detail) (DCM-d_2 (*)) of [Ru(bimca^{C5H8})Br(PPh_3)] (**5**).*

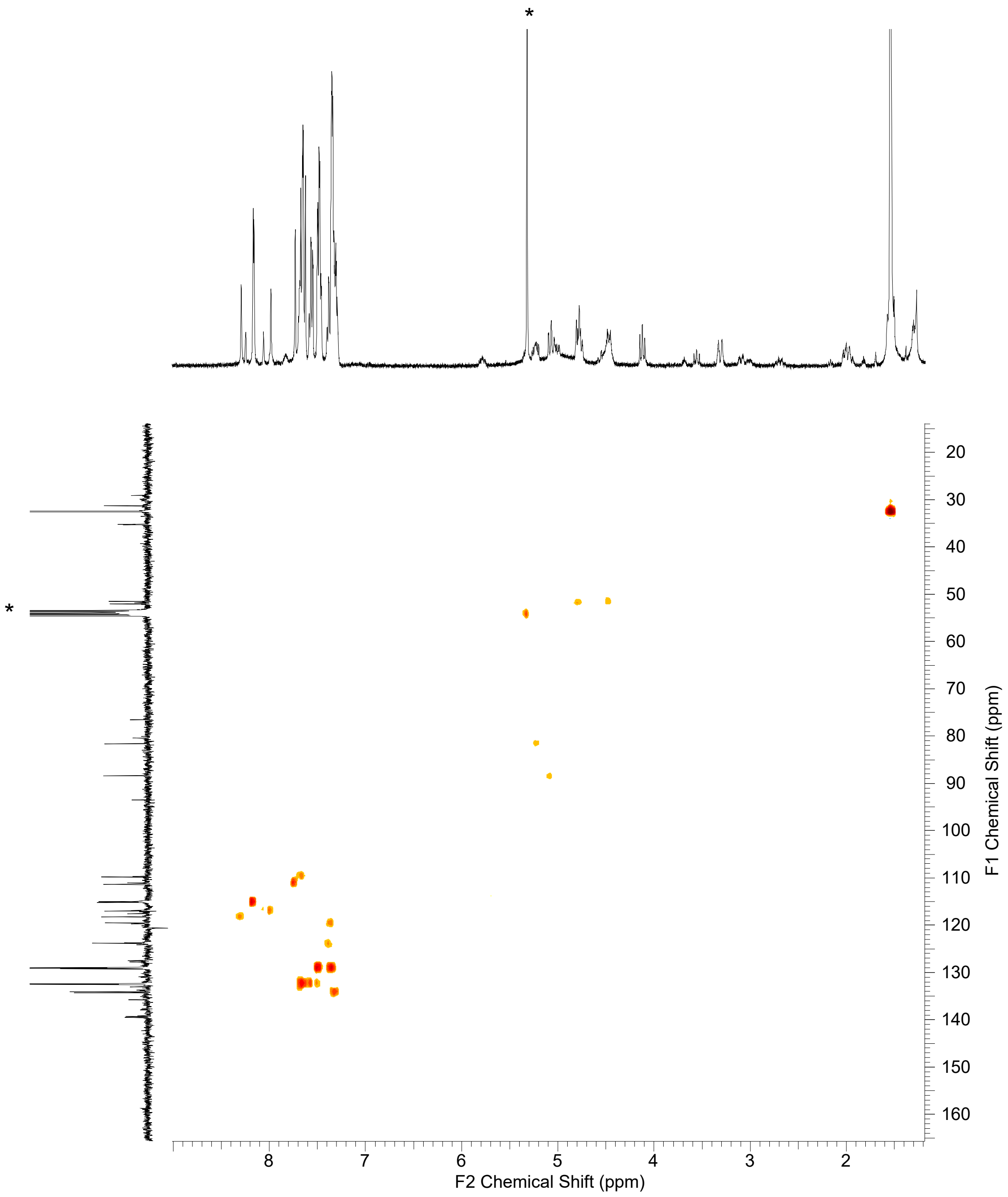

*Figure 38: $^{1}H,^{13}C$ HSQC NMR spectrum (detail) (DCM-d_2 (*)) of [Ru(bimca^{C5H8})Br(PPh_3)] (**5**).*

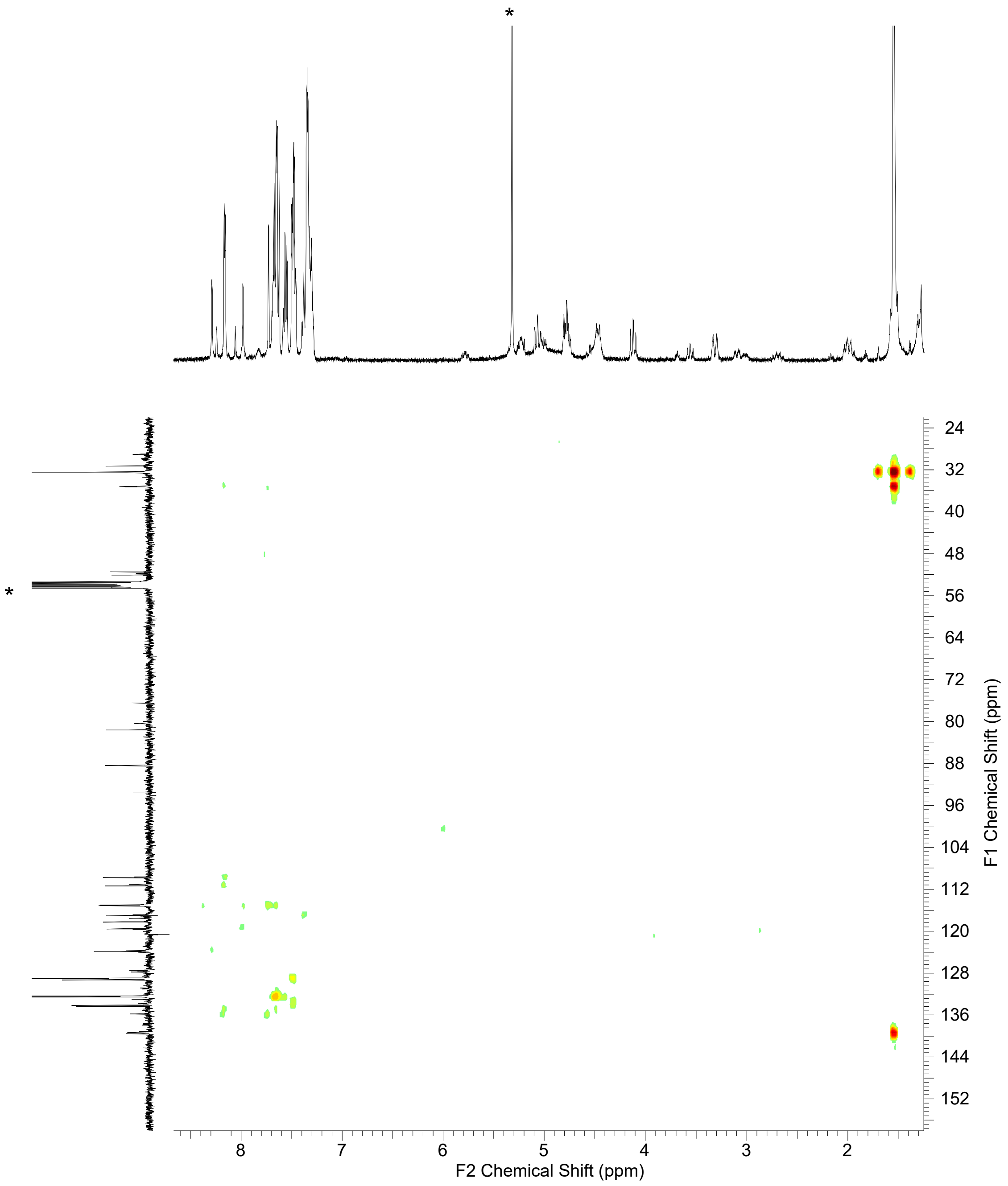

*Figure 39: ^{1}H,^{13}C HMBC NMR spectrum (detail) (DCM-d_2 (*)) of [Ru(bimca^{C5H8})Br(PPh_3)] (**5**).*

[Pd(bimca^{C5H9})] (6):

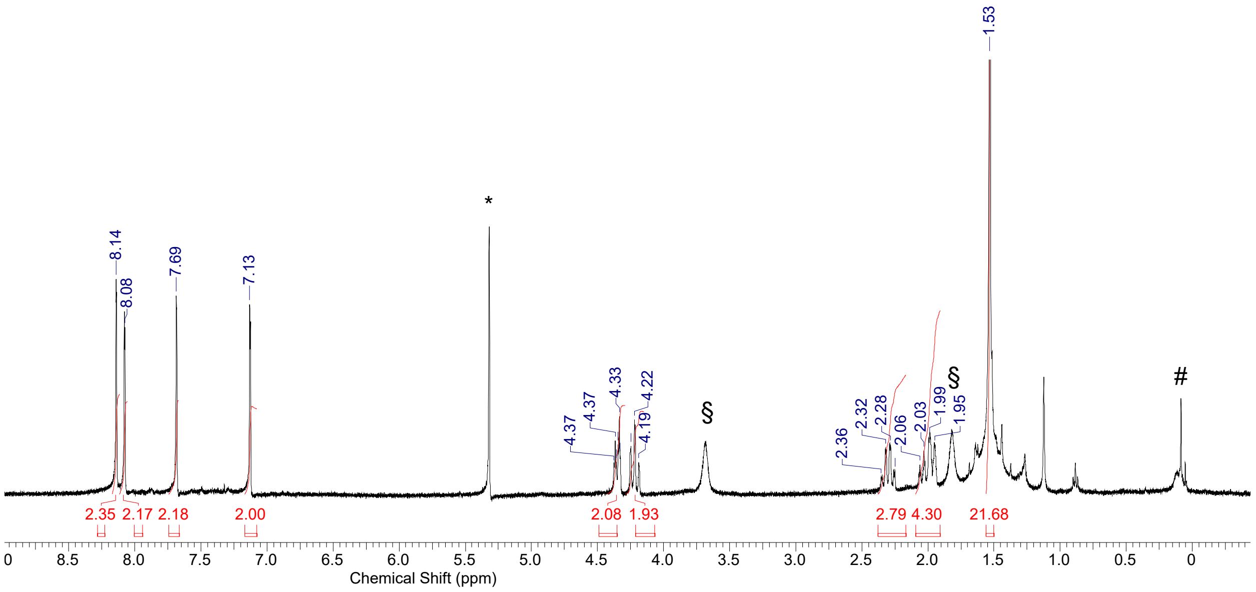

*Figure 40: ^{1}H NMR spectrum (400 MHz, DCM-d$_2$ (*)) of [Pd(bimca^{C5H9})] (**6**); grease (#), THF-d$_8$ (§).*

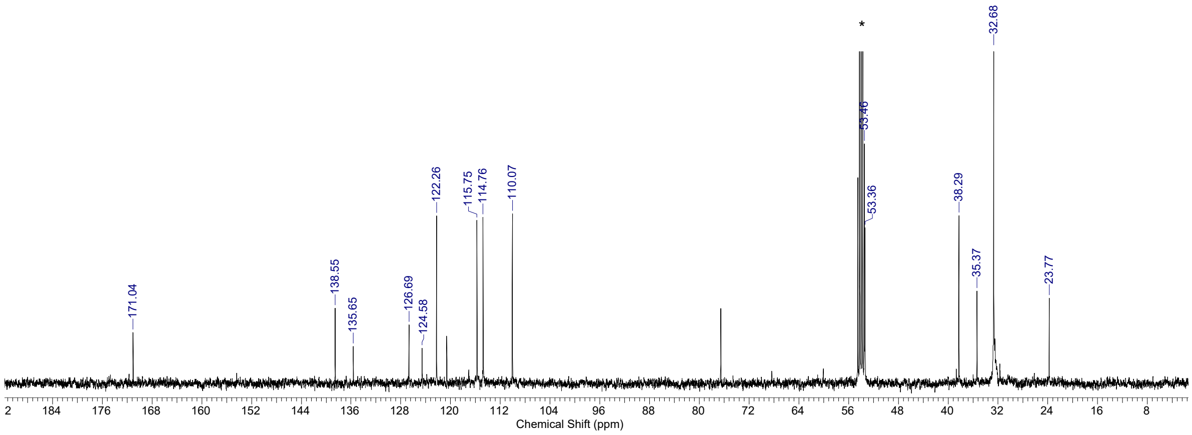

*Figure 41: ^{13}C NMR spectrum (100 MHz, DCM-d$_2$ (*)) of [Pd(bimca^{C5H9})] (**6**).*

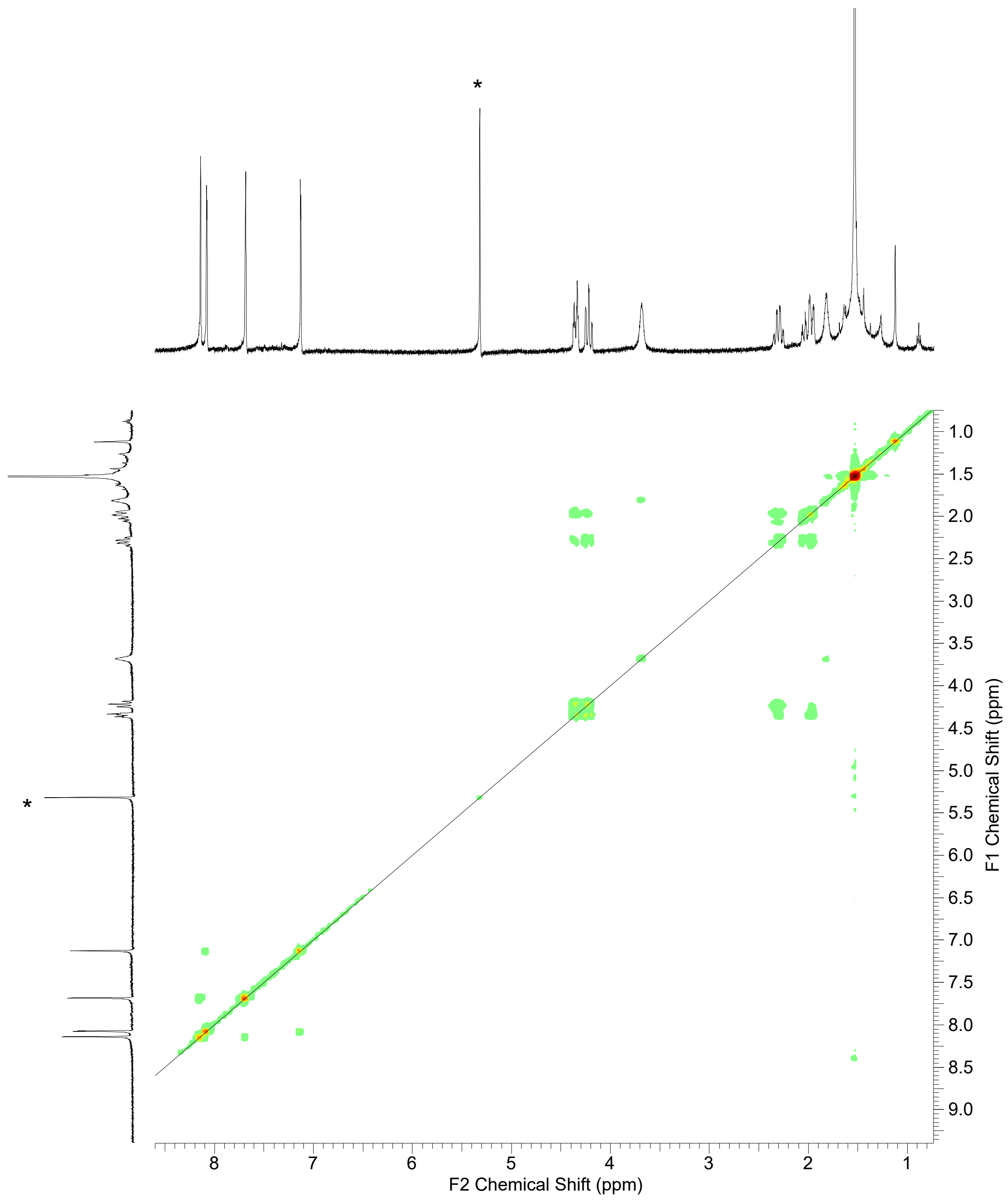

*Figure 42: $^{1}H,^{1}H$ COSY NMR spectrum (detail) (400 MHz, DCM-d_2 (*)) of [Pd(bimca^{C5H9})] (**6**).*

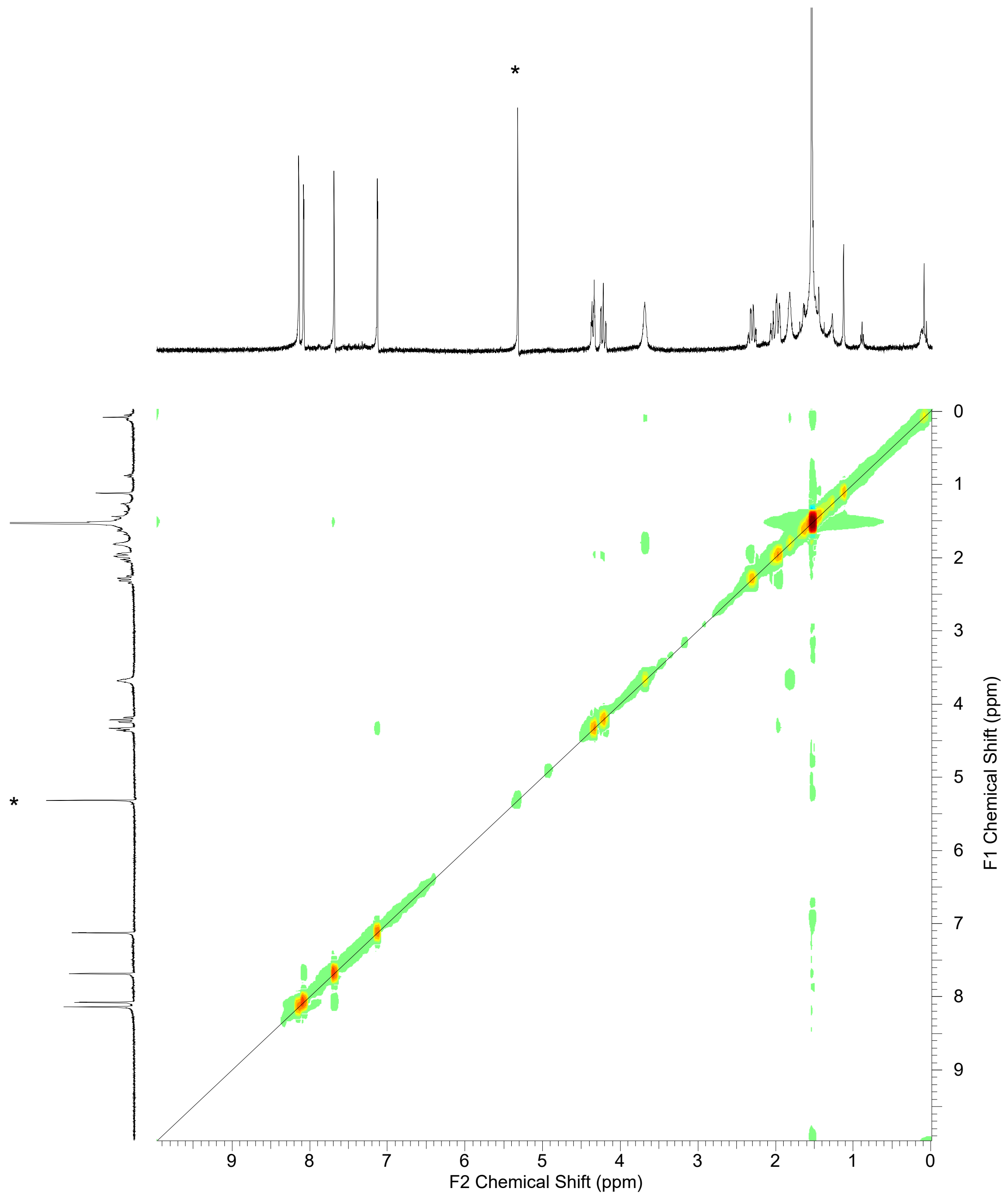

*Figure 43: $^{1}H,^{1}H$ NOESY NMR spectrum (detail) (400 MHz, DCM-d_2 (*)) of [Pd(bimca^{C5H9})] (**6**).*

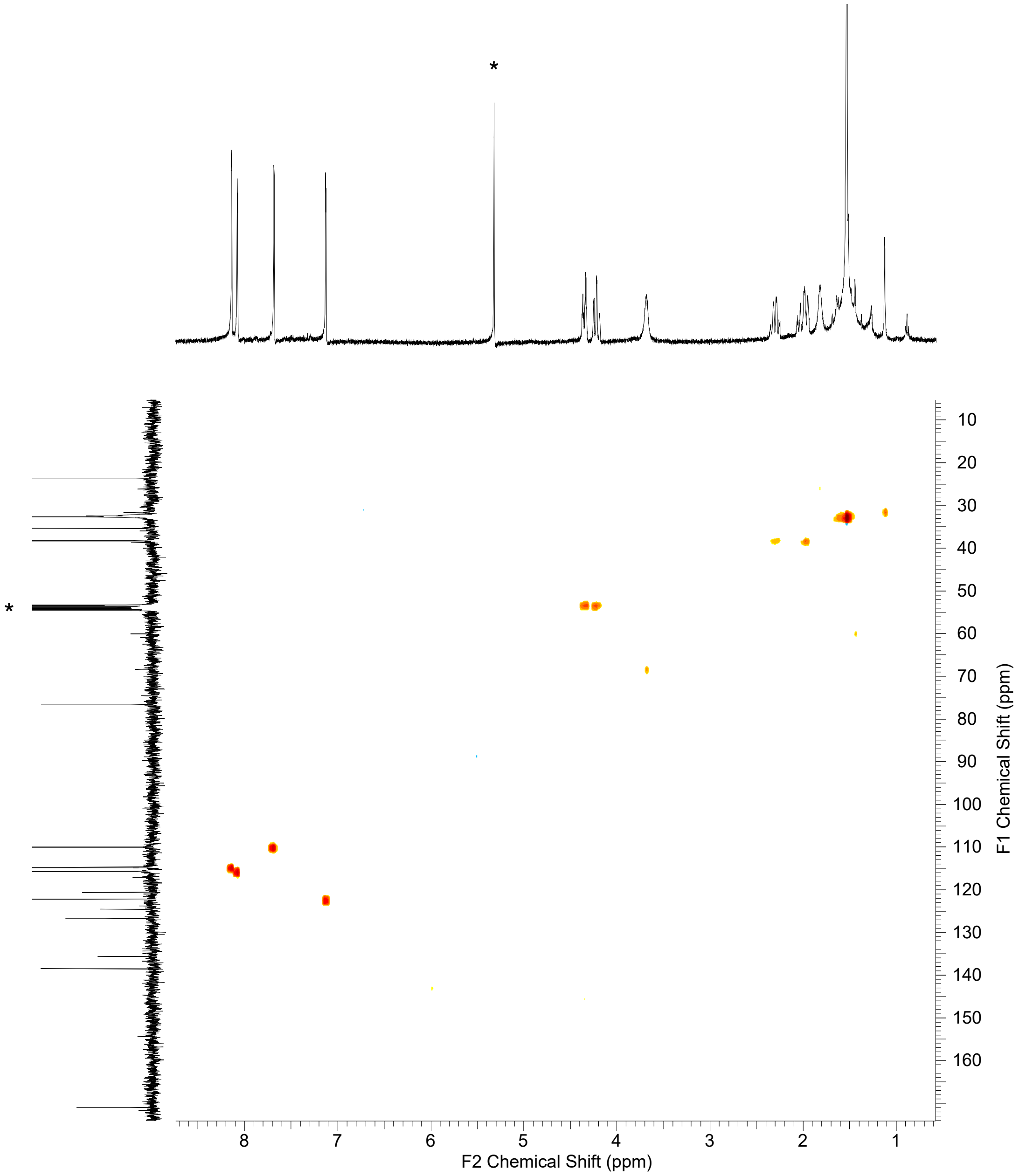

Figure 44: $^{1}H^{13}C$ HSQC NMR spectrum (detail) of [Pd(bimca^{C5H9})] in DCM-d_2 (*).

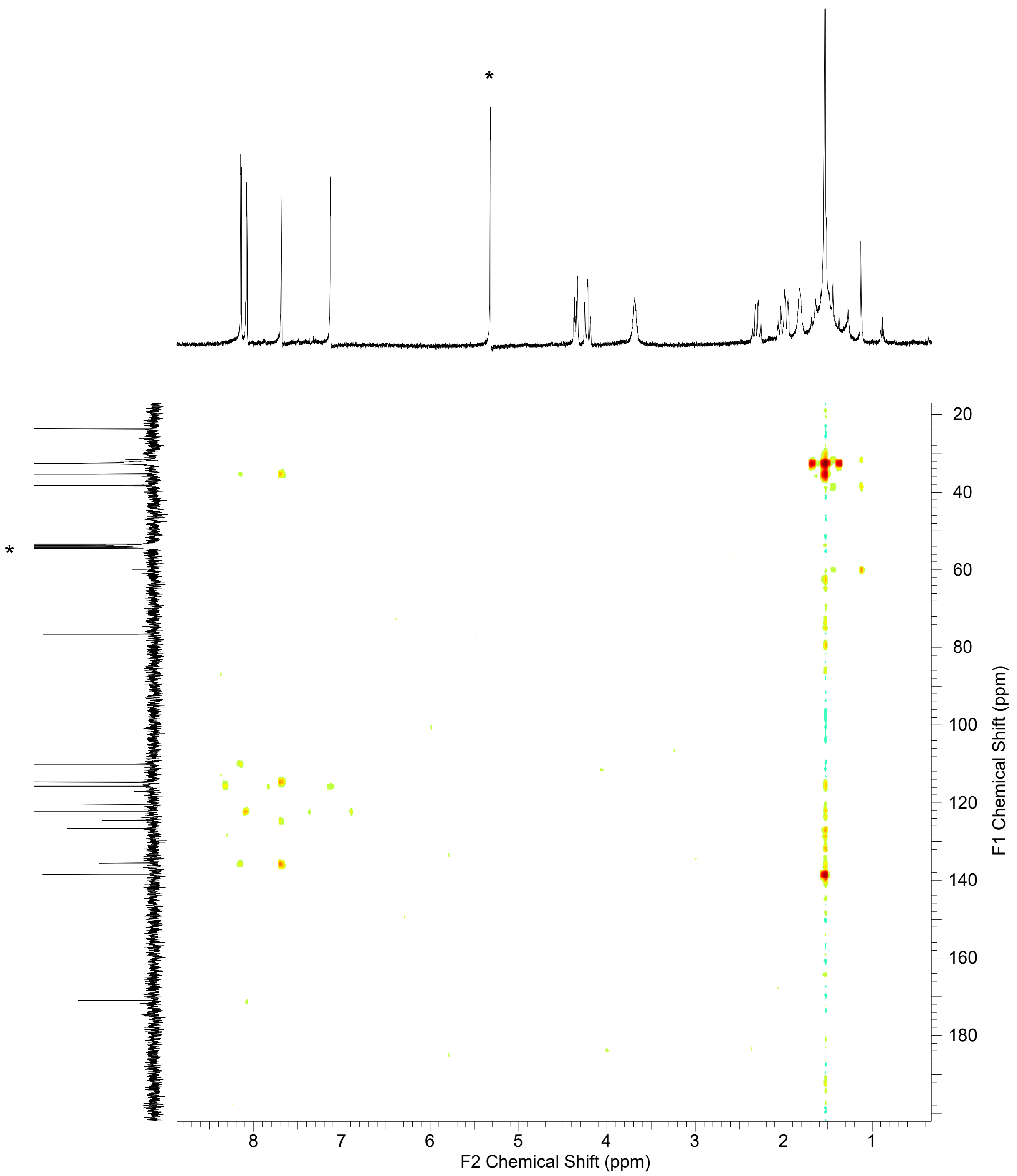

*Figure 45: $^{1}H^{13}C$ HMBC NMR spectrum (detail) of [Pd(bimca^{C5H9})] (**6**) in DCM-d_2 (*).*

2 HR-MS Spectrum of (6)

<u>**[Pd(bimca^{C5H9})]:**</u>

MS (HR-ESI$^+$, CH_3CN) calculated $[M+H]^+$ = 584.20001; measured = 584.19985 relative mass deviation = 2.27 ppm.

The experimentally obtained peak (top) is a superposition of the peaks $[M]^+$ and $[M+H]^+$ as revealed by the simulated spectra of $[M+H]^+$ (middle) and $[M]^+$ (bottom).

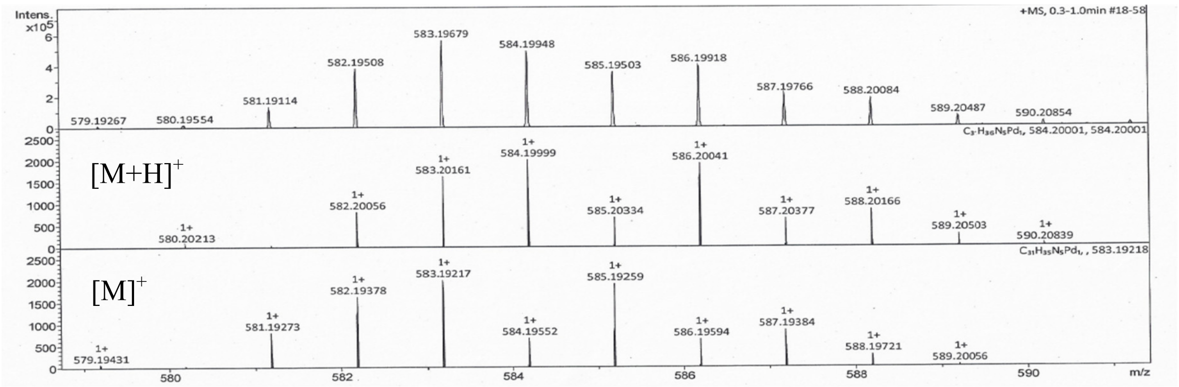

Figure 46: HR-ESI$^+$ analysis of [Pd(bimca^{C5H9})] experimental (top) and simulation of the peaks $[M+H]^+$ and $[M]^+$.

3 Crystal structure analysis

Compound	[Li(bimca^{C5})] (2)	[K(bimca^{C5})] (3)	[Ru(bimca^{C5})Cp*] (4)	[Ru(bimca^{C5H8})Br(PPh$_3$)] (5)
**CCDC No.#**	2064679	2064676	2064677	2064678
**Empirical formula**	$C_{558}H_{648}Li_{18}N_{90}$	$C_{86}H_{120}K_2N_{10}O_6$	$C_{82}H_{102}N_{10}Ru_2$	$C_{51}H_{53}BrCl_4N_5PRu$
**Formular weight**	8740.54	1468.11	1429.88	1089.73
**Temperature/K**	100(2)	100(2)	100(2)	100(2)
**Radiation**	MoK_α (λ = 0.71073)	MoK_α (λ = 0.71073)	MoK_α (λ = 0.71073)	MoK_α (λ = 0.71073)
**Crystal system**	trigonal	monoclinic	tetragonal	monoclinic
**Space group**	R-3	P2$_1$	I4/*m*	P2$_1$/*c*
**a/Å**	20.4638(5)	12.880(7)	19.800(4)	13.541(2)
**b/Å**	20.4638(5)	11.116(6)	19.800(4)	14.552(2)
**c/Å**	37.4037(10)	14.198(7)	22.655(4)	25.683(4)
***α*/°**	90	90	90	90
***β*/°**	90	95.465(10)	90	93.946(4)
***γ*/°**	120	90	90	90
**Volume/Å^3**	13564.9(8)	2023.6(18)	8882(4)	5048.8(13)
**Z**	1	1	4	4
**ρ_{calc} g/cm^3**	1.070	1.205	1.069	1.434
**μ/mm^{-1}**	0.064	0.176	0.382	1.385
**Crystal size/mm^3**	0.231 x 0.248 x 0.568	0.082 x 0.135 x 1.454	0.091 x 0.171 x 0.177	0.128 x 0.143 x 0.144
**F(000)**	4680		3008	2224
**Completeness**	99.9 %	99.5 %	99.8 %	99.3 %
**Description/colour**	plate/colorless	column/colorless	plate/red	plate/red
**Θ range for data collection/°**	2.4625 – 28.17.	2.4235 – 21.595.	2.3115 – 20.4	1.6205 – 18.61
**Index ranges**	-27≤h≤27, -27≤k≤27, -49≤l≤49		-24≤h≤24, -24≤k≤24, -28≤l≤28	-16≤h≤16, -18≤k≤18, -32≤l≤32
**Reflections collected**	81891		45658	64973
**Independent reflections**	9840 (R_{int} = 0.0407, R_{sigma} = 0.0188)		4667 (R_{int} = 0.1665, R_{sigma} = 0.0778)	10325 (R_{int} = 0.1296, R_{sigma} = 0.0904)
**Data/restraints/parameters**	9840/0/353		4667/670/367	10325/60/614
**Goodness-of-fit on F^2**	1.036		1.028	1.041
**Final R indexes R values [I>2σ(I)]**	R_1 = 0.0526, wR_2 = 0.1436		R_1 = 0.0462, wR_2 = 0.1058	R_1 = 0.0645. wR_2 = 0.1375
**Final R indeces [all data]**	R_1 = 0.0665, wR_2 = 0.1570		R_1 = 0.0815, wR_2 = 0.1200	R_1 = 0.1119, wR_2 = 0.1592
**Largest diff. peak/hole/ e Å^{-3}**	0.69/-0.41		0.44/-0.57	1.37/-0.96

4 UV-Vis and Fluorescence Spectroscopy

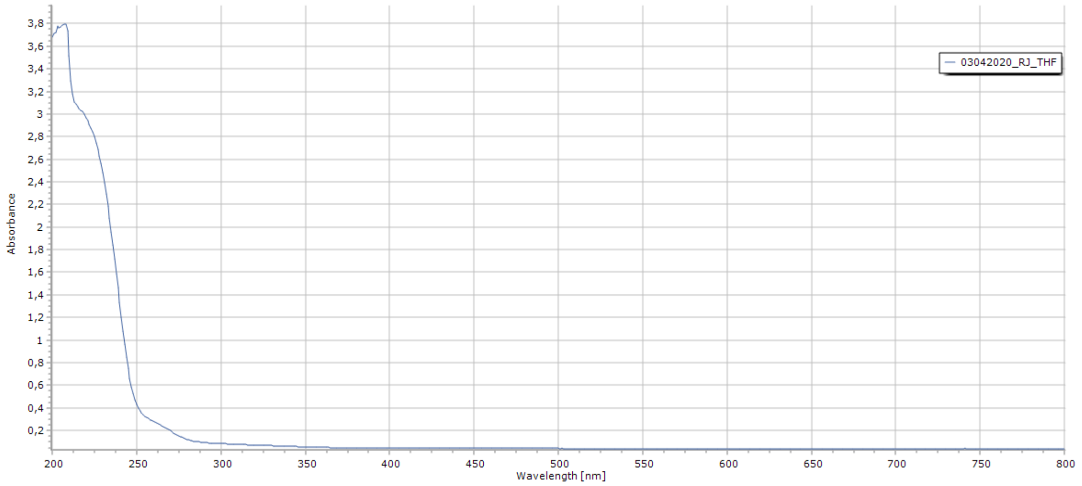

Figure 47: Absorption spectrum of THF (background)

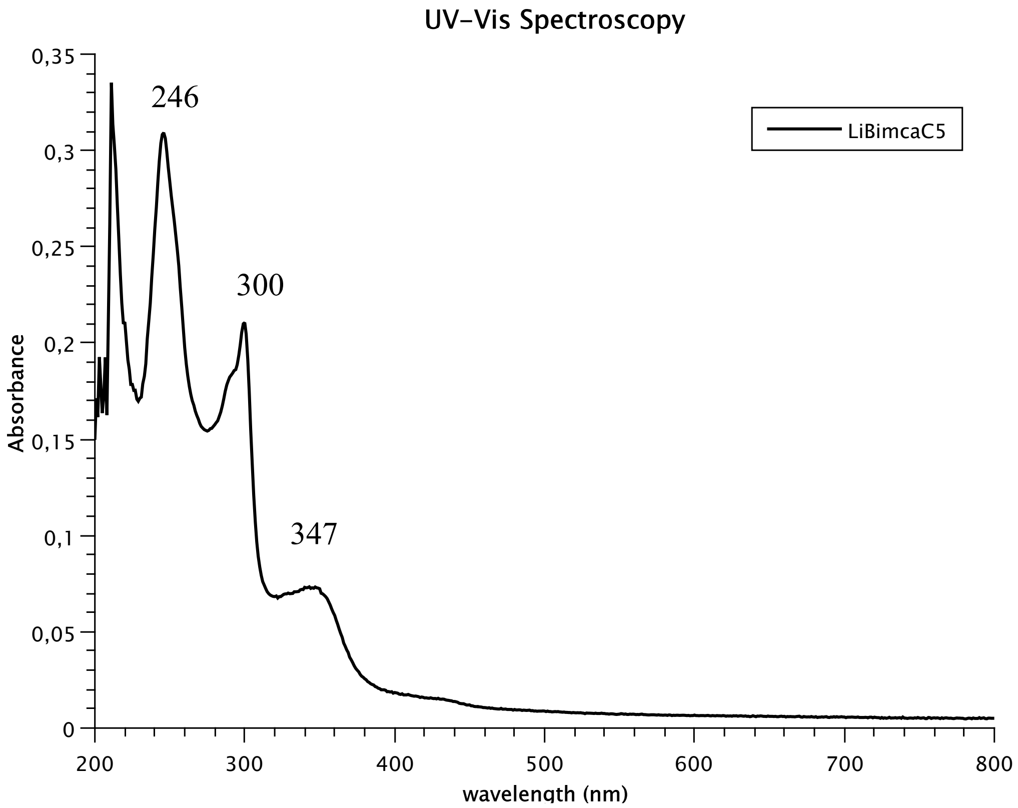

*Figure 48: UV-Vis spectrum of [Li(bimca^{C5})] (**2**) (Tetrahydrofuran, c=: 4 * 10^{-5} M).*

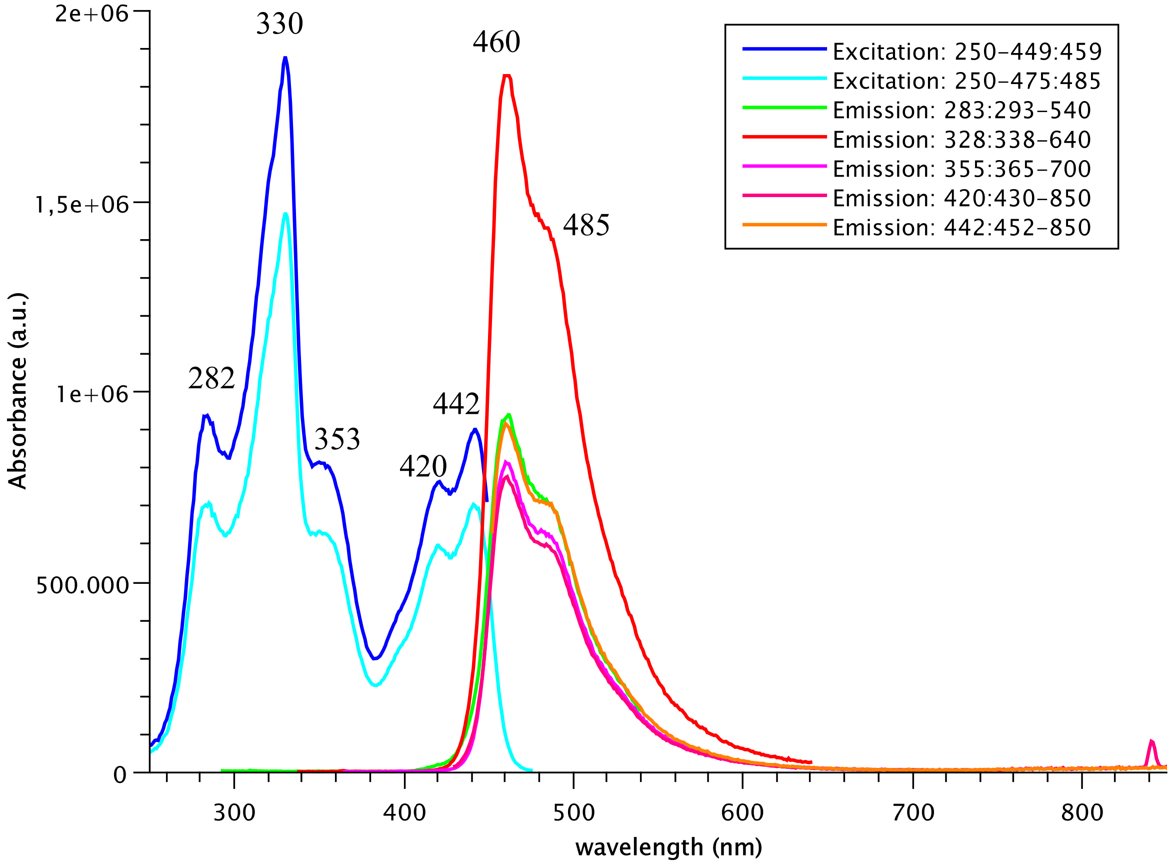

*Figure 49: Excitation and emission spectra of [Li(bimca^{C5})] (**2**) in solution. (Tetrahydrofuran, c = 4 * 10^{-3} M).*
